# The future of Forkhead box O transcription factors

**DOI:** 10.1042/BCJ20253428

**Published:** 2026-02-03

**Authors:** Huanjie Huang, Tianshu Gui, Boudewijn MT. Burgering

**Affiliations:** 1 Center Molecular Medicine and Oncode Institute, University Medical Center Utrecht, Utrecht, Netherlands

**Keywords:** aging, Forkhead proteins, insulin signaling, intrinsically disordered proteins, signal transducers and activators of transcription, transcription factors

## Abstract

The Forkhead box O (FOXO) class of transcription factors is evolutionary conserved both structurally and at least in part also functionally. FOXO activation results in transcriptional programs that provide cellular resilience toward exogenous and endogenous challenges, especially challenges that provoke cellular oxidative stress. This FOXO-dependent mechanism of resilience explains by and large the observed longevity phenotype in model organisms where increased FOXO activity extends lifespan. This may even hold for human lifespan as genome-wide association studies show a strong linkage between FOXO3 and human lifespan. Despite decades of studies on FOXOs, there are still many unresolved questions. Here, we discuss some of these knowledge gaps, related to our general understanding of transcriptional control by FOXOs, the role of the intrinsically disordered regions that constitute over 50% of FOXOs sequence, the role of cellular context in determining isoform specificity, and finally, the importance of resilience in understanding FOXO function. The latter, we think, provides context to the evolutionary role of FOXOs. So, rather than providing an exhaustive summary of literature findings, we focus on some of the omissions in our knowledge of FOXO function. Resolving these outstanding questions, we think, will help to provide the necessary insight to know how and when to manipulate FOXO function in a manner that will contribute to healthy aging.

## Introduction

Forkhead box O (FOXO) transcription factors (TFs) constitute a subfamily of the larger family of Forkhead domain TFs, that are characterized by a conserved 80–100 amino acids DNA-binding domain (DBD) known as the forkhead box or winged helix. In mammals, the O-class consists of four isoforms: FOXO1, FOXO3, FOXO4, and FOXO6.

**Figure 1:**
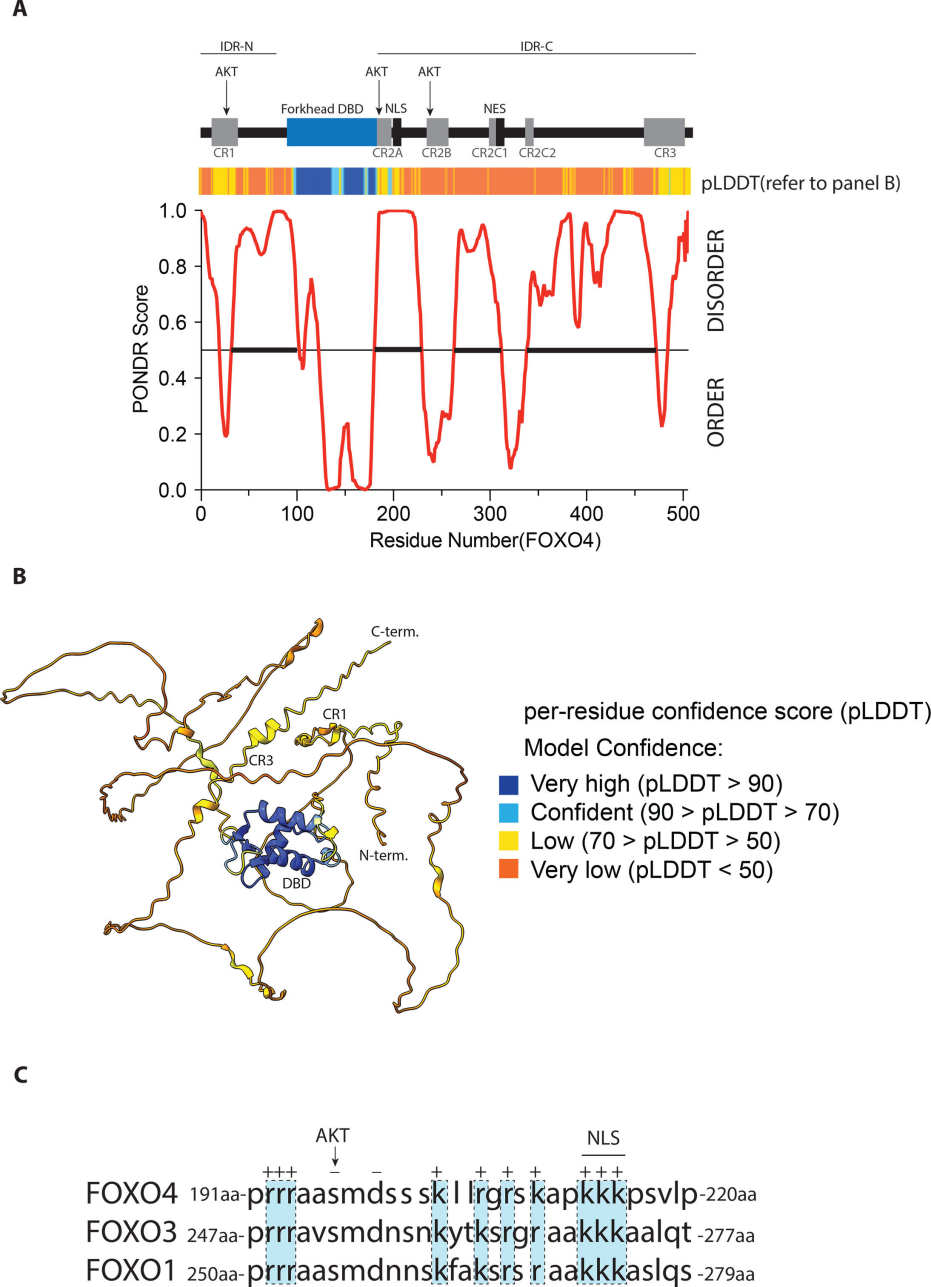
Domain organization of FOXOs (A) The N- and C-terminal IDR flanking the DBD are indicated (IDR-N and IDR-C). Within the IDR, the SLiMs that are conserved between FOXO members are indicated and these conserved regions have propensity to fold [6–9]. These regions encompass besides the forkhead domain the 3 PKB/AKT phosphorylation sites (for reference these are within FOXO3 Thr32 located in CR1, ser253 within CR2A and ser315 within CR2B), the NLS and nuclear export signal the latter being part of the CR2B, two lysine rich domains (CR2C1 and CR2C2) involved in the histone acetyl transferases CBP/p300 binding(178) and finally the transactivation domain at the end of the C-terminal domain (CR3) which is implicated in binding various coregulators. The PONDR prediction is aligned with the domain cartoon above indicating that indeed the CR regions are predicted to have a propensity to form structure compared with the rest of the IDR. (B) Alphafold model of FOXO4: We used the human FOXO4 sequence in Alphafold2. The color scheme represents the per-residue confidence score (pLDDT) between 0–100. Regions with pLDDT below 50 can be considered unstructured in isolation. The 3D structure prediction intuitively presents the structured region and the IDRs within the protein. Although the model shown here is for FOXO4, the alphafold prediction for FOXO1 and FOXO3 are highly similar. For orientation purpose the N- and C-terminus as well as the DBD and CR3 and CR1 are indicated. (**C**) The CR2A domain is highly negatively charged. ClustalW alignment of CR2A region of FOXO1,3 and 4. Positive charges are indicated as well as the PKB/AKT phosphorylation site and the adjacent negatively charged amino acid residue. This region has a Net charge of 9 at pH 7.0. and this region is preceded by a proline in all isoforms suggesting this region can participate as ‘extended’ DBD and add in search and capture of FOXO DNA binding sequences.

Basically, three important observations have sparked interest in understanding FOXO function. First, FOXOs have been described as direct substrates of protein kinase B (PKB) [1–3]. PKB is homologous to the serine/threonine protein kinase encoded by the oncogene in the transforming retrovirus isolated from the thymoma cell line AKT-8, hence the alternative name AKT [4]. PKB/AKT kinase, and therefore FOXO, is controlled through phosphoinositide 3-kinase (PI3K) signaling [5]. PKB/AKT-mediated phosphorylation of FOXOs occurs on three different conserved amino acid positions (FOXO4 [1], FOXO3 [2], FOXO1 [3], see Figure 1A). Phosphorylation by PKB/AKT leads to translocation of FOXO from the nucleus to the cytosol and hence inhibits FOXO transcriptional activity [2]. PI3K mutations as well as genetic deletion of the phosphatase and tensin homolog (PTEN), the negative regulator of PI3K, are commonly observed in many different types of human tumors. In agreement, in various model systems, FOXOs regulate cellular processes that are intrinsically linked to tumorigenesis (e.g., cell cycle progression, cell death, metabolism). PI3K/AKT drives cell proliferation, whereas active FOXOs, in agreement with being inhibited by PKB/AKT-mediated phosphorylation, inhibit cell proliferation. Thus, in this context, FOXOs can be considered as tumor suppressor genes.

Second, during development toward adulthood, the nematode *Caenorhabditis elegans* can enter a stress-resistant, dormant stage (the Dauer larva) in response to harsh conditions like starvation, allowing them to survive these adverse environments for extended periods before resuming normal development when conditions improve. Genetic dissection of Dauer formation identified a network of Dauer regulating genes (coined DAF), among which activation of DAF-16 following loss of DAF-2 activity prolongs lifespan [10]. DAF-2 is the orthologue of insulin/IGF-1 receptor and DAF-16 is the orthologue of FOXO. Subsequent genetic studies demonstrated that DAF-16 function is controlled by PI3K/AKT signaling in a manner identical to what is observed for FOXO in human cells [11]. Consequently, it was shown that also genetic manipulation of PI3K (AGE-1 in *C.elegans*) [12] and PTEN (DAF-18 in *C.elegans*) [13] signaling similarly results in lifespan changes [14]. Furthermore, genome-wide association studies showed that various single nucleotide polymorphisms located within or near the FOXO3 gene locus showed a strong and importantly reproducible linkage with increased human lifespan [15]. Together, these findings show that even human lifespan, although formally not subject to Darwinian genetic selection, can be affected by gene activity and this started a whole field of new (scientific) activity to search for ways to intervene in lifespan, genetically or non-genetically.

Third, whereas PI3K/PTEN regulation of FOXO links FOXO to cancer, the observation that in *C. elegans* this pathway acts downstream of the insulin/IGF paralog DAF-2, links DAF-16/FOXO also to insulin signaling. In agreement, deregulation of PI3K signaling toward FOXO is strongly implicated in diabetes (reviewed in [16,17]), linking DAF-16/FOXO not only to the aging process but also to two major age-related diseases, namely cancer and diabetes.

The number of reviews on FOXO is manyfold and sometimes unavoidable and reiterative. Consequently, rather than providing another summation of literature, we here provide an attempt to point out possible relevant blind spots in our knowledge concerning FOXO function, resolution of which we think may be helpful to further our understanding of FOXO’s role in cellular signaling and organismal health.

### Aging and the role of FOXO

Aging is the natural process of time-dependent functional decline of an organism. This decline in function is not uniform but segmental; it occurs at all levels within an organism, from cell to organ but with different kinetics. This variability in decline at all levels has several consequences. First, when making the distinction between aging and lifespan (longevity), organismal lifespan is limited by aging, yet not all organs are equally relevant in this respect. An extreme example is the graying of hair which has no known impact on life expectancy. Yet aging of the heart will limit lifespan as it increases the chance of death by heart failure. Second, the timeline of aging can be variable between individuals and does not per se correlate with chronological age. This poses the need to define and measure biological age, as compared with chronological age, preferably in a manner that links critical processes of aging to this biological clock. Several studies have defined biological clocks to accommodate the notion that the level or speed of decline is not linear. These clocks have been developed by comparing molecular markers like histone acetylation, serum protein composition, and others to discriminate between biological age and chronological age. Examples of clocks defined in this way are epigenetic clocks, most notably the Horvath clock [18,19], but also more tailored clocks like GrimAge [20], and clocks based on other cellular parameters like proteomic clocks [21], and clocks based on inflammatory markers (iAge) [22].

These aging clocks can provide an estimate of an individual’s biological age, and importantly, these clocks can be influenced by factors like lifestyle and environment. By measuring biological age, these clocks can help to assess disease risk, evaluate the effectiveness of interventions, and potentially identify factors that contribute to aging. However, at present, all these clocks likely represent only part of the picture (see [23] for discussion).

Although a decline in function does not necessarily result in disease onset, it certainly will increase chance (denoted as frailty in aging). Diseases are limiting lifespan, but a life without disease is limited in time. As aging is determined by intrinsic and extrinsic challenges that cells, organs, and an organism face, it is oftentimes suggested that the ability to withstand aging is a function of resilience. Resilience is frequently used interchangeably with adaptability and coping, and we will use these terms interchangeably here. Resilience can be attributed to cells and organs and specific signaling pathways that function by providing cells and organs with coping strategies. Interestingly, resilience is not restricted to the molecular biology domain, and, for example, social resilience may also contribute to increasing lifespan, as suggested for the observed longevity in the Okinawa population [24].

Nowadays, it has been shown that in model systems like *C. elegans,* almost up to 30% of all genes can have an impact on longevity. However, in many cases, gain or loss of function gene manipulation(s) results in shortening of lifespan, whereas for the same gene, the reverse manipulation only in a limited number of examples increases lifespan. FOXO/DAF-16 was the first gene to display reduced lifespan in case of loss of function and an increase in lifespan in case of gain of function. Importantly, of all genes associated with longevity in model organisms, FOXO3 is one of two, the other being apolipoprotein E (ApoE), consistently found to be associated with human longevity [25]. Multiple human GWAS studies across different human populations have linked single nucleotide polymorphisms (SNPs) at the FOXO3A locus to human longevity [15]. Importantly, all these SNPs are located outside the FOXO3 coding region and thus do not result in an amino acid alteration that may explain a change in FOXO3 function. The effect of these types of SNPs can be on the level of FOXO3 expression, or alternatively, these SNPs may change the differential expression between FOXO3 splice variants. For example, Santo et al. described a FOXO3A transcriptional isoform FOXO3A-Short (FOXO3A-S), encoding a major longevity-associated SNP, rs9400239 within its 5' untranslated region [26]. The rs9400239 variant influences the stability and functionality of the primarily nuclear protein(s) encoded by the FOXO3A-S mRNA. Expression of the shorter variant, in turn, affected glucose clearance in agreement with suggestions that handling of glucose is a major determinant of human lifespan [27,28]. In contrast with FOXO3, SNPs in FOXO1, FOXO4, and FOXO6 do not appear to be as strongly linked to human longevity [29,30]. Although, there is evidence of a joined effect on longevity of FOXO3 and FOXO1 SNPs [31], which may be gender specific [29]. In any case, the strong association with FOXO3 [26] already poses the question of what is different between FOXO3 and the other isoforms.

Bioinformatic analyses of FOXO DNA sequences suggest that vertebrate FOXO genes originate from successive gene duplications [32]. Gene duplication allows the occurrence of genetic alterations to acquire novel function in one of the two paralogues as the other paralogue can retain its original function [33]. However, FOXO members show clear functional redundancy illustrated by the observation that only triple floxed knock-out mice (FOXO1, 3, and 4) show increased incidence of tumorigenesis, and premature mortality attributable to vascular lesions was observed only in mice with somatic deletion of all three FOXOs [34]. Furthermore, also outside the conserved DBD, the regions for PKB/AKT phosphorylation and regulation of co-factor binding are also highly conserved (see further on); thus, FOXO function may be more of a ‘family affair’ (see discussion in [35]). This could argue that the specific relevance of either FOXO isoform may be mostly determined by its tissue-specific expression. Being this the case, we still must consider in what specific details FOXO members may differ as different tissue likely provides different context for exerting shared functions.

Given the above, the question becomes imminent how the different FOXO isoforms regulate transcription, and what is shared and what is specific to the different isoforms? How then does transcriptional regulation translate at the level of regulating gene transcription? Indeed, in mice, broad somatic deletion of all FoxOs as opposed to deletion of individual FoxO alleles caused a progressive cancer-prone condition characterized by thymic lymphomas and hemangiomas [34]. This same study showed that genetic deletion of FoxO in lung endothelial cells compared with liver endothelial cells resulted in a differential gene regulation where, e.g., FoxOs control Sprouty2 gene expression in liver but not in lung endothelial cells. These observations clearly demonstrate that in molecular terms, signaling downstream of FoxOs is both cell type-specific and tissue-specific within the same cell type, and thus, FoxO regulation of these targets *in vivo* is highly context-specific, even in the same cell type. This functional redundancy and context specificity raise the question of how functional specificity is achieved in different cellular contexts despite their shared DNA-binding capacity.

The first compelling explanation came from work by Sellers and coworkers [36] that demonstrated a FOXO mutant lacking DNA-binding ability could still exert tumor suppression and cell cycle regulation by modulating a subset of target genes, likely through protein–protein interactions with other transcription factors. This indicates that FOXOs may function through non-canonical mechanisms by forming complexes with lineage- or context-specific transcriptional partners, thereby regulating gene expression without direct DNA binding. Indeed, emerging evidences have shown that FOXOs are able to associate with diverse TFs, resulting in diverse transcriptional outputs in different tissues (reviewed in [37]). Association with other TFs can be direct or mediated by DNA in cases where DBE of FOXO is adjacent to another TF consensus sequence. Thus, the genomic organization, the cellular presence of certain TFs as well as their activity, regulated through signaling pathways, provide the context that combined determines whether FOXOs co-operate with certain TFs and regulate specific gene transcription.

Second, others and we have shown that certain TF co-activators, specifically β-catenin [38–40] and Yes-associated protein (YAP) [41,42], can be tethered away from their cognate TFs, TCF and transcriptional enhanced associate domain (TEAD), respectively, following cellular oxidative distress. This co-activator switching mechanism, therefore, results in FOXO activation and concomitant down-regulation of TCF and/or TEAD.

Third, FOXOs have been described as lineage-restricted [34] and many FOXO target genes as well as FOXO isoforms exhibit tissue-specific expression patterns [43]. A recent review [43] used data from the Human Protein Atlas (HPA) (www.proteinatlas.org) to generate an inventory of FOXO isoform mRNA and protein expression in human tissues. mRNA expression of FOXO1, 3, and 4 is by and large ubiquitous although levels may vary considerably. FOXO1 is the prevalent isoform in the thyroid, liver, endometrium, ovary, skeletal muscle, smooth muscle, lymph node, and tonsil. FOXO3 is the predominant isoform at the transcript level in the retina, parathyroid, lung, salivary, esophagus, tongue, stomach, colon, duodenum, rectum, small intestine, gallbladder, pancreas, kidney, bladder, prostate, vagina, breast, cervix, adipose tissue, skin, bone marrow, appendix, spleen, and thymus. FOXO4 is the most expressed FOXO transcript in the brain, adrenal gland, testis, epididymis, seminal vesicles, and placenta [43].

Unfortunately, in general, there is a low concordance between mRNA and protein expression [44], and this also may account for FOXOs. Combined with the generally poor, or at least varying, quality of antibodies commercially available for FOXOs, it should be concluded that FOXO isoform protein expression is still mostly uncharged territory. Whereas expression of, e.g., FOXO1 and FOXO3 is observed in many cell types, phenotypes are oftentimes attributed to either one of these isoforms. An example being the role of FOXO3a, but not FOXO1 or FOXO4, which was essential for Interferon-γ (IFN-γ) induced class II major histocompatibility complex (MHC II) gene expression in macrophages and was recruited as a component of the MHC II enhanceosome upon IFN-γ treatment [45]. While this may be the case, it should be noted that experimental evidence in this respect should be considered carefully. First, FOXO3 has been shown to regulate FOXO1 expression [46], and consequently, a FOXO3 knockdown will concomitantly reduce expression of FOXO1. Second, FOXOs induce complex feedback signaling, the best described example being mTORC2-mediated PI3K/AKT activation and consequent inhibition of FOXO activity [47,48]. Thus, ectopic expression of, e.g., constitutive active FOXO3 mutant will reduce activity of FOXO1 and FOXO4, suggesting that formally the observed phenotype could be due to the other isoforms rather than FOXO3. Third, as for many TFs, it is unclear whether FOXOs act both as transcriptional activator and repressor. Evidence indicates that FOXOs act predominantly as transcriptional activators [49,50], so gene repression is in most cases a secondary/indirect event and will be likely dependent not only on the nature of the transcriptional repressor regulated by FOXOs but also on the level of induction. This confounds interpretation of down-regulated gene signatures after FOXO activation.

Finally, context can also determine the outcome even when FOXO activation results in transcriptional regulation of identical genes. FOXOs are mediating resilience (see also further on); however, this function itself could be considered agnostic for context. Consequently, we have argued previously that mediating resilience in an untransformed cell prevents transition to a diseased state, most notably a transformed cancerous cell. However, in a cancer cell context, the same function of FOXO, namely mediating resilience can contribute to increased cancer cell survival. Thus, depending on the context, namely normal cell versus cancer cell, FOXOs, through the same downstream transcriptional regulation, can act as tumor suppressor or tumor-promoting gene (see for extended discussion [51]). A different example is provided by the observation that FOXO1 repressed the maintenance of early small intestinal intraepithelial tissue-resident memory CD8 T (Trm) cells in contrast with its actions in sustaining Trm cells from small intestinal lamina propria and colon and contrary with its broader role in promoting intestinal Trm cell formation [52].

Therefore, although FOXOs compensate for each other to a certain extent, their transcriptional output may still vary depending on the cellular lineage or tissue type, and as argued above [51], even the same functional output may have different consequences. Such context-dependent activity may be shaped by differential expression patterns, co-factor availability, chromatin accessibility, or isoform usage across distinct cell types. This implies the utility of integrated transcriptomic and epigenomic analyses may contribute to reconstructing the regulatory networks of TFs.

Nevertheless, literature shows a large variety in FOXO-associated ‘gene signatures’. So far, there is not a single FOXO target gene whose expression can reliably serve as a universal indicator of FOXO activity across all cell types and tissues or in response to all stimuli. However, when defining evolutionary conserved DAF-16/FOXO target genes, it becomes clear that some target genes appear more reliable in the sense that these show up in many but not all signatures [53]. In addition, it becomes clear that FOXOs regulate many cellular processes (metabolism, cell cycle progression, cell death) that provide resilience to a variety of challenges (e.g., redox changes, DNA damage, nutrient changes, infection), yet the specificity of genes involved is less conserved and mostly context (type of challenge) dependent. These context-dependent transcriptional programs reflect the ability of FOXOs to integrate environmental cues with cellular needs, suggesting that FOXO-mediated resilience is shaped by a combination of isoform-specific functions, cofactor interactions, and chromatin accessibility. To understand the latter, some critical questions need to be addressed: What defines transcriptional output besides the DBD? Can transcriptional output be isoform specific or solely depending on tissue-specific expression of the different FOXO isoforms?

### How do FOXOs function as TFs?

The above provides some rationale as to how FOXOs may act as context-specific transcriptional regulators, but additional molecular mechanisms can be at play, and these are less explored. Previous research has shown that particularly FOXA1 but also FOXA2, other family members of the larger Forkhead box TFs, display high nonspecific nucleosome binding affinity both *in vitro* and *in vivo* [54–57]. This specific function that discriminates FOXA from TFs that cannot interact with condensed nucleosomes led to the identification of FOXA as a ‘pioneer factor’. A pioneer factor is defined as being able to engage silent, closed chromatin, to initiate the recruitment of additional factors that aid in opening of condensed chromatin, and thereby activate/open regulatory DNA sequences for subsequent binding of TFs, ultimately leading to specific biological functions (reviewed in [58]). The ability to interact non-specifically with nucleosomes has been attributed to the similarity in 3-D structure between the Forkhead domain and histones, specifically H5 [59]. Consequently, the Forkhead domain could exchange with histones and incorporate into nucleosomes without major structural consequences. In line herewith, FOXO1 has been also reported to be able to bind nucleosomes and to de-condense compacted chromatin arrays *in vitro* [60,61]. It is not yet known whether *in vivo* FOXO1 acts as a pioneer factor and whether the other FOXO isoforms can also act as a pioneer factor. This question would be relevant to answer because by acting as pioneer factors, FOXOs would enable other TFs to exert their function. This could explain how FOXO activation may give rise to a diverse array of gene expression patterns, as this would in part also depend on the activity (or not) of other TFs, making FOXO gene expression signatures highly context dependent. These observations also align with studies that show FOXO interaction with other TFs either directly or by co-binding onto the DNA through adjacent binding motifs, like Suppressor of Mothers against Decapentaplegic (SMAD) [62], Early region 2 binding factor (E2F1) [63], Forkhead box G1 (FOXG1) [62], Foxo1-CoRepressor (FCoR) protein [64], Zinc finger protein 238 (Zfp238) [65], and even p53 [66] for extensive listing (reviewed in [37]).

When bound to DNA or nucleosomes, TFs recruit modifying enzymes to open the DNA, to stimulate formation of an RNA polymerase transcription initiation complex or to stimulate transcription elongation. In the case of FOXOs, little is known as to how FOXOs contribute to these different aspects of transcription. FOXOs bind to p300/CBP via specific protein–protein interaction domains, and this results in histone acetylation and FOXO acetylation. Nucleosomal histone acetylation may be sufficient to recruit a preinitiation complex containing RNA polymerase II to the gene promoter. Subsequently, acetylation of sites near the DBD attenuates its DNA-binding ability [67]. Together, this indicates acetylation-mediated turnover of FOXO DNA binding to regulate transcriptional output. Several studies have investigated the importance of TF DNA-binding turnover (residence time) in transcriptional output, and although it is likely too early to generalize outcome, it appears that long residence time compared with fast turnover determines output [68]. In this respect, Chip-seq, being a single time point snapshot of a highly dynamic system, does not capture dynamics and hence does not predict transcriptional output.

In addition to p300/CREB binding protein (CBP), there is surprisingly little known on additional chromatin remodeling complexes/enzymes that may interact with FOXOs. In *C. elegans*, DAF-16/FOXO recruits the chromatin-remodeling complex SWItch/sucrose non-fermentable (SWI/SNF) to promote stress resistance and longevity [69], yet in mammalian cells, this has not been addressed specifically. However, interaction between FOXO and the ATPase brahma-related gene 1 (BRG1) has been suggested, albeit that it is unclear whether this interaction is direct [70]. BRG1 is a subunit of the BRG1/BRM-associated factor (BAF) complex that belongs to the SWI/SNF- remodelers. In addition to SWI/SNF, an interaction with SIN3a has been suggested [71], and SIN3a is part of the REST-CoREST repressor complex [72]. Finally, bromodomain-containing protein (BRD4) binds to enhancer regions and combines with positive transcription elongation factor b (P-TEFb) to regulate transcription elongation. In agreement with the observation that at least FOXO3 regulates gene transcription through enhancer regions [73], acetylated FOXO3a has been shown to recognize the BD2 domain of BRD4. However, it should be noted that BRD4 is an ‘epigenetic reader’ in contrast to SWI/SNF and others that are chromatin modifiers/remodellers. The potential involvement of other remodeling complexes, like nuclear receptor co-repressor (NcoR), Nucleosome Remodeling Deacetylase) complex (NurD), Imitation swItch (ISWI) [69], in FOXO-controlled gene transcription remains unknown and deserves attention.

### Understanding the role of FOXOs intrinsically disordered regions

**Figure 2:**
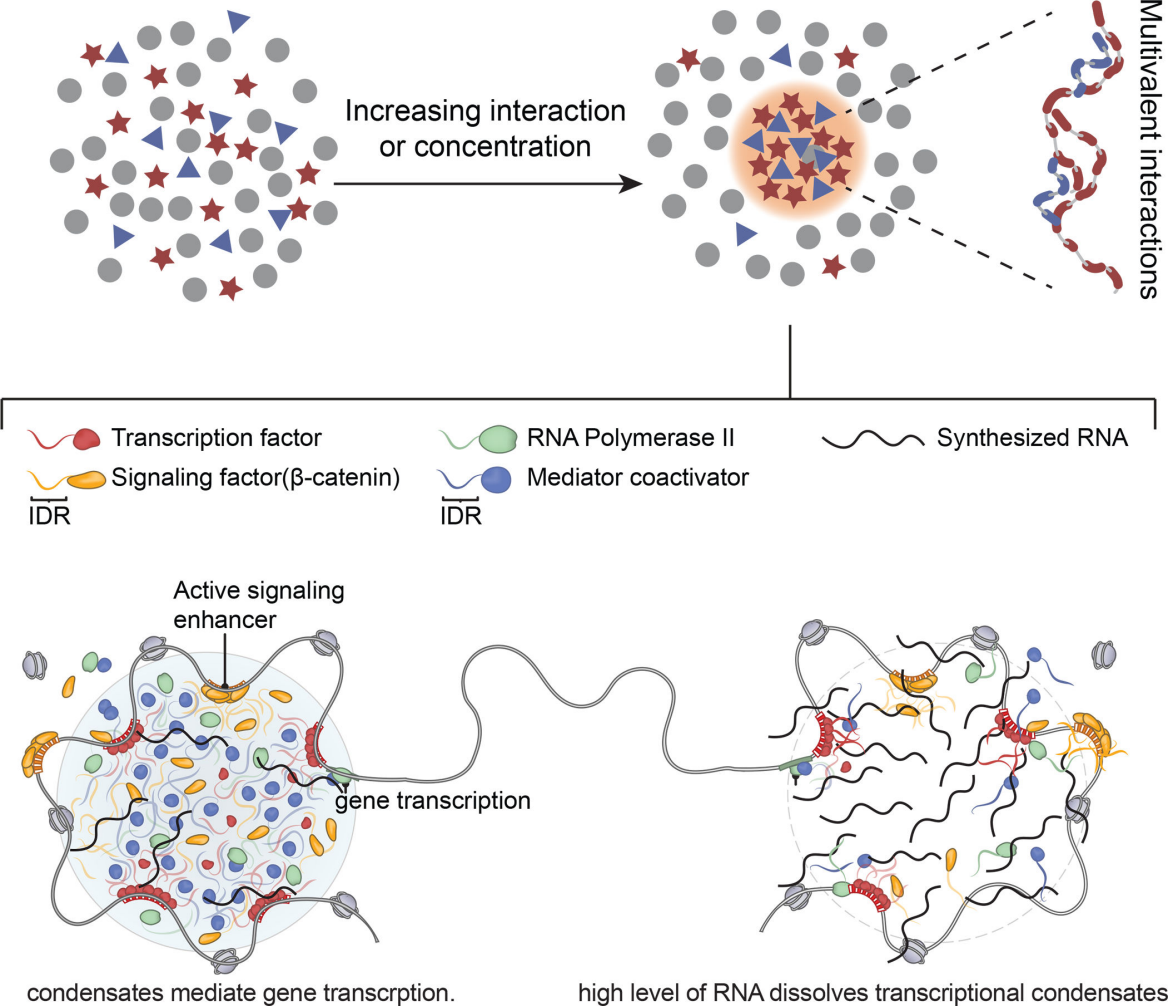
A summary of biocondensate formation and its role in transcription regulation. Cellular content is seen as a homogeneous mixture of protein, lipid, RNA and DNA. To become optimally functional, biomolecules need to organize, and compartmentalization is a major organizing principle. Condensate formation is one example as to how a compartment can be established. Proteins associate through multivalent interactions, thereby increasing concentration and excluding other proteins that are not functional in a given process, e.g., transcription. In case of transcription, condensation is triggered by TFs engaging in multivalent interactions with other transcription regulating proteins like MED1 and BRD4 (see main text). Likely TFs, because of their ability to selective bind DNA regions, determine at what genomic locations condensates are formed. Because of the 3D structure of the genome, it is suggested that within one condensate multiple TF binding sites are brought into vicinity, establishing a transcription hot spot, in which multiple genes can be transcribed in a concerted manner. Finally high concentrations of mRNA can dissolve condensates and hereby this acts as a negative feedback mechanism.

In most earlier studies, understanding FOXO function and regulation focused on the role of posttranslational modifications as a means to recruit co-factors/regulators and the DBD. However, recently it has become clear that the N- and C-terminal domains flanking the DBD are regions that mostly lack clear or fixed 3D structure, and these are now commonly referred to as intrinsically disordered regions (IDRs). In general, the conformation of proteins is dynamic, and in certain cases, there is no single dominant conformation under physiological conditions (reviewed in [74]). Especially, proteins that contain IDRs can sample a large range of conformations due to the intrinsic plasticity of the unstructured segments (Figure 2). Due to this conformational disorder, specific functions are attributed to IDRs. In the case of FOXOs, we will briefly discuss the N- and C-terminal IDR functioning as a platform for (-) a diverse array of post-translational modifications (PTMs), (-) for binding co-factors and (-) as possible allosteric regulator of FOXO function and (-) as possible search and capture module for DNA binding.

IDRs are challenging to study because they can confer specificity despite low sequence complexity and can be functionally conserved despite rapid sequence divergence. However, IDRs harbor short linear motifs (SLiMs), which are sequences of 3–12 amino acids with a propensity to fold into an α-helical structure especially upon binding to other proteins. In general, SLiMs can be classified into two primary categories: ligand motifs, which facilitate binding interactions as scaffolding modules, and post-translational modification (PTM) motifs, which are recognized and altered by modifying enzymes [75]. Therefore, through SLiMs, IDRs are often an entry point of control by signaling pathways, because signaling through specific PTMs (phosphorylation, acetylation, ubiquitination) regulates binding partner choice and hence involvement in cellular processes [76].

In FOXOs, several SLiMs can be identified, and in contrast to the expected rapid evolutionary drift of IDRs, these are conserved regions between FOXOs and consequently denoted conserved region 1–3 (CR1, CR2A, CR2B, CR2C1, CRC2, and CR3 see Figure 1A). In a recent study called the Critical Assessment of Protein Intrinsic Disorder prediction (CAID), 32 disorder prediction methods and 11 prediction methods that detect binding motifs within IDRs were evaluated [77]. This systematic benchmarking study provides valuable guidance for selecting the most appropriate disorder prediction algorithm. Here, we used Predictor of Natural Disordered Regions (PONDR) [78] to predict the probability of disorder based on amino acid composition of FOXO proteins (Figure 1A and B). Moreover, AlphaFold2 and its updated versions have emerged as prominent tools for predicting the 3D structures of proteins and, by omission, IDRs [79]. AlphaFold-Multimer, another version of the software, can even predict the formation of protein complexes, providing insights into the behavior of IDRs and their interactions with other molecules [80]. These computational tools, combined with experimental methods, have significantly advanced our understanding of IDRs. For each FOXO isoform, AlphaFold indeed yields a predicted structure that clearly shows the large extent of disorder flanking the DBD (Figure 1B). This observation underscores the possibility of IDRs in FOXOs as a key structural basis for the context-dependent functionality of FOXO proteins.

### IDRs—a platform for modification and binding co-factors

IDRs are prime targets for post-translational modifications (phosphorylation, acetylation, ubiquitination). Within ordered 3D structures, amino acid residues that are potentially subject to modification can be buried within the structure and therefore inaccessible for modifying enzymes. Because of the structural flexibility, this does not account for IDRs, and PTMs are overrepresented within IDRs compared with structured regions. Consequently, many PTMs have been reported for FOXOs within the N- and C-terminal domain flanking the DBD. Within the N- and C-terminal IDR of FOXOs, several small regions have been identified that show propensity to form an α-helix, and interestingly, these domains show highest conservation between FOXO isoforms (CR1 in the N-terminal domain, CR2a, CR2b, CRC1, CRC2, and CR3 within the C-terminal IDR (see [81] for review). These conserved regions are involved in binding of co-factors, and some of the PTMs are located within these conserved regions and contribute to regulation of co-factor binding. Also, some PTMs are in the conserved regions and then are mostly observed for all isoforms, whereas outside the conserved regions, there appears to be more isoform specificity in terms of PTMs. However, it must be noted that the identification of PTMs is not always unambiguous. Although mass spectrometry can identify specific modified residues, the certainty of identification is not always definitive and sometimes requires additional confirmation. This is also illustrated by the observation that when comparing the ability of proline-directed kinases, e.g., cyclin-dependent kinases (CDKs), stress-kinases (p38, JNK), NLK, and ERK, many of the same residues can serve as phospho-acceptor sites for these kinases, suggesting either considerable redundancy or a requirement for more rigorous testing. Also, compared with FOXO3 and FOXO1, FOXO4 is likely underrepresented in whole phosphoproteome mass-spectrometry studies because of its absence or low expression level in many cell types. Trypsin digestion, commonly used to generate peptides that can be analyzed by mass spectrometry, does not provide equal coverage of peptide identification for the different isoforms, resulting in regions for which PTM information is missing. This may explain why, e.g., for FOXO3, several PTMs are described between residues 40 to 190, whereas these are lacking in FOXO1 and FOXO4, except for one random phosphorylation site (S153 in FOXO1 and S111 in FOXO4). Whereas many possible PTMs are identified for FOXOs, for many, the stoichiometry at which these PTMs occur is unknown. This is relevant to know as due to the unstructured nature of the C- and N-terminal region of FOXOs, it is likely that many PTMs occur randomly and may represent noise in signaling.

FOXOs are responsive to oxidative distress [82]. Initially, we and others have shown that stress kinases like Jun kinase (JNK) that are activated upon oxidative stress phosphorylated FOXOs and regulated cytosolic-nuclear shuttling [83–85]. However, changes in cellular redox state also result in specific and reversible cysteine oxidation in target proteins. Cysteine residue oxidation can lead to a range of post-translational modifications, one of which is the formation of inter and/or intramolecular disulfides. We have shown that FOXO3 and p300/CBP interact through a cysteine disulfide bridge [86] and importantly that this covalent interaction can be reduced and thus is reversible. As the cysteine residues in FOXOs are located within the respective IDRs, this provides an alternative mode to generate stable interactions without the need of structure. Furthermore, we showed that the different cysteines with the FOXO isoforms are used to differentially bind co-factors [87]. The nuclear import receptors Importin-7 (IPO7) and Importin-8 (IPO8) form a disulfide-dependent heterodimer with FOXO3, which is required for its reactive oxygen species-induced nuclear translocation. Yet FOXO4 does not interact with IPO7 or IPO8 [87]. Whereas this illustrates that differences in primary amino acid sequence within IDRs can determine specificity of cofactor binding between the different FOXO isoforms, it remains that all FOXOs undergo nuclear-cytoplasmic shuttling. By using different partners to achieve the same, e.g., kinetics of shuttling may differ. However, it is unknown how this may affect transcriptional output.

Although the DBD is highly conserved, amino acid differences between FOXO isoforms within the DBD may alter affinity toward DNA and/or DNA binding kinetics. This may affect the level of transcriptional output (see discussion further on), but to our knowledge, there are no studies that show this to determine isoform-specific function. On the same note, the IDR regions of the FOXO isoforms lack high sequence conservation, the defined conserved regions within the IDRs (Figure 1A), but again, we are not aware of studies that indicate a specific amino acid change in these conserved regions to be involved in acquiring a specific isoform function. Nevertheless, there are amino acid differences that impose differential upstream regulation. Notably, FOXO3 is phosphorylated and regulated by IKKbeta on a FOXO3 specific serine residue (ser644) located in CR3 [88]. However, this and other findings of isoform-specific regulation are relevant to explain why FOXO3 and not other FOXOs may be regulated in a specific context, but on the other hand, this does not provide evidence on possible isoform-specific outcome.

### IDRs and binding DNA

In addition to the above, IDRs in TFs may contribute to DNA binding [89]. The structured DBDs are frequently immediately flanked by IDRs, rich in positively charged amino acids (lysine, arginine, or histidine). In most instances, the flanking IDRs together with structured DNA-binding region are defined as integrated DBDs, since they specifically interact with the major groove of target DNA. In other cases, the positively charged flanking IDRs can intrude the minor groove through nonspecific charged interactions, as the minor groove primarily consists of the negatively charged phosphate backbone [90]. In both scenarios, IDRs promote TF’s DNA-binding affinity through interactions with DNA specifically or nonspecifically. Indeed, within FOXOs, the IDR immediately following the C-terminal end of the DBD is conserved (see Figure 1C) and harbors a stretch of positively charged amino acids (K, R). Interestingly, this region also contains two SLiMs. First, the sequence for PKB/AKT phosphorylation (second site) and second the NLS sequence. Phosphorylation adds negative charge, followed by the conserved negatively charged amino acid. Together, this may neutralize the positive charges and thereby impair DNA binding. This PKB/AKT-mediated weakening of DNA binding, in turn, may facilitate the interaction between FOXOs and 14-3-3. In addition, lysine acetylation in this region is conserved between isoforms [91,92] and may shield charge of this region. Indeed, CBP/p300-mediated acetylation has been shown to weaken FOXO DNA interaction *in vitro* and is suggested to thereby facilitate PKB/AKT phosphorylation and consequent release from DNA and relocation to the cytosol [67]. This role of the IDR and the PTMs is in line with that described for other TFs like Sox2 [93], Hox proteins [93,94], and LEF-1 [95]. Although further studies are needed, these similarities at least suggest that FOXOs also use this extended DBD module for DNA binding. In addition, this suggests the NLS to act both in mediating nuclear location and indirectly nuclear exit. However, given the sequence conservation of this extended DBD region, it is unlikely to explain possible isoform differences in full.

### IDR-mediated biomolecular condensation

IDR-mediated biomolecular condensation, caused by phase separation, is another emerging mechanism that may contribute to FOXO-regulated transcription. Phase separation is a process where a homogeneous mixture separates into two distinct phases, typically a dense liquid phase and a dilute liquid phase in the case of liquid-liquid separation (Figure 2) and [96]. An increasing number of studies demonstrate that phase separation, driven by proteins or nucleic acid polymers that self-associate, leads to the formation of ‘biomolecular condensates’ in the cell [96–98]. Importantly for biological systems, the molecules inside the dense phase are both internally dynamic and exchangeable with the dilute phase [97,99].

Phase separation is increasingly recognized as a mechanism to organize various cellular compartments. Biomolecular condensates are membrane-less compartments that include P granules [100], nucleoli [101], and heterochromatin domains. They have been referred to by various descriptive terms, such as cellular bodies, puncta, dots, granules [98,102].

Multivalency is crucial for condensate formation, and multivalent molecules have increased tendency to assemble into large oligomers or polymers. At thresholds of concentration and affinity, this assembly process overcomes the solubility of the molecules due to entropy-driven effects, leading to phase separation [103]. As indicated, IDRs provide intrinsic plasticity to proteins, allowing them to adopt a range of conformations, and the SLiMs present in IDRs can act as scaffolding elements and provide multiple weakly adhesive motifs for both intra- and intermolecular interactions, which can promote condensation [98]. Furthermore, PTMs enriched within IDRs can lead to the alteration of interactions, subsequently affecting phase behavior. For instance, phosphorylation within the N-terminal IDR of Fused in Sarcoma (FUS) has been found to reduce FUS phase separation [104].

With respect to transcription regulation, a recent study demonstrated that condensates mediated by the IDR of Mediator complex (MED1) selectively partition RNA polymerase II together with its positive allosteric regulators while excluding negative regulators, in a charge-pattern-dependent manner [105]. This study supports a model wherein condensates selectively partition molecules that combined execute function, e.g., transcription activation (Figure 2).

By now, examples of biomolecular condensate formation have been described for many different proteins involved in transcriptional regulation, including TFs [106,107], co-factors [108], mediator components [109,110], RNA polymerase II [110,111], and nucleic acids [109,112]. A recent study has shown that TF FoxA1 can bind to DNA and subsequently mediate TFs-DNA condensation *in vitro* [113]. Further studies demonstrate that the transcription condensates in cells are enriched at super enhancers [106,109].

Subsequently, TF-DNA condensates recruit other transcriptional components, such as Mediator of RNA polymerase II transcription subunit 1 (MED1) [109], transcriptional coactivators like Bromodomain-containing protein 4 (BRD4) [109], and RNA polymerase II [110,111,114,115]. The assembly of transcriptional condensates leads to burst of RNA production and subsequently amplification of gene expression [116]. Interestingly, it was also reported that high levels of RNA synthesized at active genes can dissolve condensates, suggesting RNA-mediated feedback control of transcriptional condensates (Figure 2) [112].

The transcriptional co-factor β-catenin also forms condensates *in vitro* and *in vivo* [108]. Wingles-related integration site (WNT) signaling inhibits proteasomal degradation of β-catenin, and this results in shuttling of β-catenin from cytosol to nucleus and binding to TCF. In agreement, activation WNT signaling increases nuclear β-catenin condensate formation, and we recently demonstrated that transcription factor 7L2 (TCF7L2) can bind to DNA alone but can form larger condensates with DNA when in the presence of β-catenin [117]. Also, β-catenin condensates recruit other transcriptional regulators like BRD4 and MED1 [109]. β-catenin also interacts with FOXO [38,118], and all FOXO isoforms can form condensates *in vitro* [117]. Whether *in vivo* FOXOs also co-localize with β-catenin in condensates is unknown.

Although met with initial skepticism [119], it is becoming clear that condensate formation represents a key step in transcription regulation. Therefore, this warrants further detailed studies to investigate how condensate formation contributes to FOXO-mediated transcription regulation. In addition, as the IDRs of the different FOXO isoforms are most divergent in sequence and are essential in condensate formation, studying condensate formation and composition by the various isoforms may also inform on possible isoform specificity.

### FOXO, where and when?

**Figure 3:**
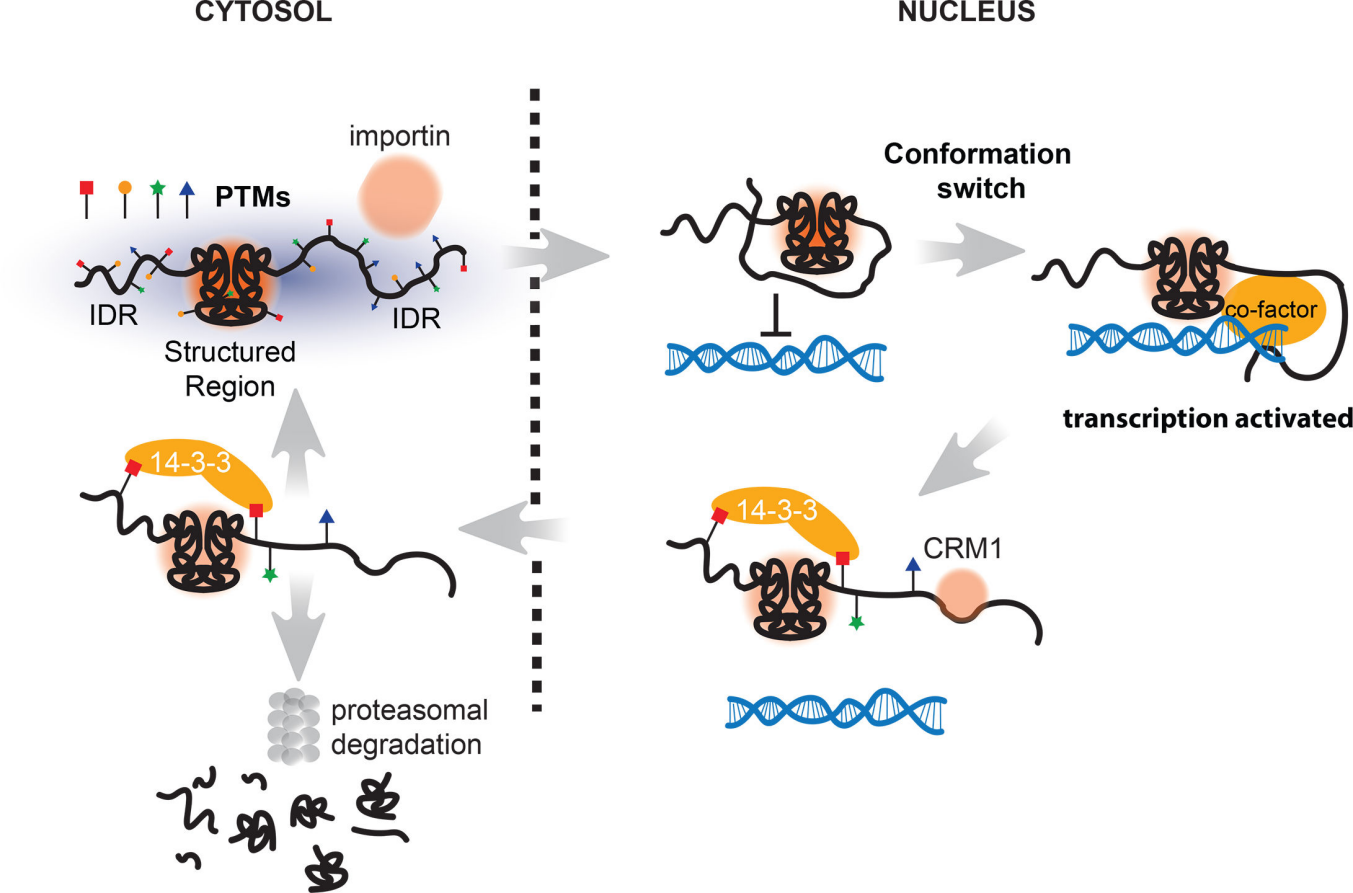
conformational dynamics of FOXOs Because of the flexible nature of the N- and C-terminal IDR regions, FOXOs can interact with a number of co-regulators using similar domains. For nuclear entry FOXOs need to interact with importins [87], within the nucleus FOXOs can adopt an auto-inhibited conformation because the CR3 domain can fold back onto the DBD [67]. This conformation competes with conformations in which FOXOs are bound to co-regulators like β-catenin or p300/CBP (see for discussion [6]). Phosphorylation and acetylation in particular at and around CR2A loosen DNA binding and eventually trigger cytosolic relocation [67]. Within the cytosol FOXOs can engage in recycling back to the nucleus or in case of poly-ubiquitination become degraded by the proteasome (see for discussion [120]).

FOXOs represent one of many TFs that is known to actively shuttle between nucleus and cytosol, and the initial notion that PKB/AKT-mediated phosphorylation resulted in cytosolic localization and hence transcription inhibition appeared to be sufficient to understand the dynamics of FOXO regulation. For one, this resulted in the reasoning that nuclear localization of FOXOs equals FOXOs being active as transcription regulators. However, as discussed above already, the dynamics of DNA binding are complex and, at least for FOXOs, not well understood. In addition, we showed that FOXOs exist in different conformations (based on binding co-factors like, e.g., 14-3-3 and β-catenin) within the nucleus [6] and (Figure 3). Thus, nuclear location is a prerequisite for transcriptional activity, but there is a definite need to better understand the dynamics of the conformational changes to fully grasp how FOXOs regulate transcription. Also, the notion of nucleo-cytoplasmic shuttling questioned whether there is a non-nuclear, cytosolic, function for FOXOs. In this respect, studies identified a role for cytosolic FOXO in autophagy (discussed in [121]) and the possibility that FOXO, i.e. FOXO3 may translocate to mitochondria and may regulate transcription within mitochondria (reviewed in [122]). Clearly, FOXO nucleo-cytoplasmic shuttling cannot be simplified as a FOXO on-off mechanism, and more research is needed to understand the consequence(s) of FOXO dynamics and whether this may represent or contribute to isoform specificity, as basically nothing is known on different dynamics of FOXO isoforms.

### FOXO-mediated resilience

Common external challenges for organisms are fluctuations in temperature, nutrient availability, and radiation (UV, daylight fluctuations). Cells, organs, and organisms must cope with these challenges, yet the strategies to do so may differ. A challenge oftentimes induces a state transition that allows one to survive the challenge, and when the challenge subsides, the state transition is either reversible (homeostasis) or irreversible (e.g., senescence).

In *C. elegans,* DAF-16 was originally identified as one of several genes that affect Dauer formation. *C. elegans* develops through a series of distinct stages: an embryonic stage, followed by four larval stages (L1-L4), and finally, adulthood. In response to unfavorable conditions, such as overcrowding or limited food, *C. elegans* can enter a Dauer larva stage, a specialized third larval stage. This allows survival for extended periods until conditions become favorable again. In this respect, Dauer is similar to what is coined Torpor, which is defined as a state of decreased physiological activity, usually marked by a reduced body temperature and metabolic activity. Torpor, thereby, is an adaptive, protective state, engaged by a range of mammals and birds, in response to, among others, aforementioned challenges (reviewed in [123]). Torpor is like hibernation, although torpor is usually induced by acute environmental changes, while hibernation is more controlled as it is preceded by a period of excessive feeding before entering the low metabolic state that accompanies hibernation. Yet, Torpor and hibernation share features like a reduction in metabolic rate [124,125]. Also, the changes in metabolism and gene reprogramming that underlie diapause and hibernation are similar [126]. Thus, torpor, Dauer, and hibernation in a similar manner all provide resilience and enable animals to tolerate food scarcity and environmental hardship, conferring a survival advantage.

FOXOs induce a G1 cell cycle arrest, and importantly, this arrest is accompanied by inducing transcription of PI3K regulators that establish a feedback mechanism whereby PI3K/AKT signaling is activated. Thus, the FOXO-induced G1 arrest is intrinsically wired to be timely, and through this feedback, cells will eventually re-enter the cell cycle (reviewed in [127]). Thus, by analogy, it could be argued that FOXOs can establish a coping strategy alike torpor at the cellular level. This possibility is further corroborated by the observation that DAF-16/FOXO responds to temperature changes. Heat shock [128] as well as cold shock [129] induces DAF-16/FOXO nuclear translocation. Furthermore, FOXOs are also responsive to changes in nutrient availability, for example, after nutrient deprivation through AMP-activated protein kinase (AMPK)-mediated phosphorylation (ref [130] and reviewed in [131]). In addition, the control of FOXO function through insulin/PI3K/AKT directly links FOXO to conditions of nutrient (glucose) excess.

The importance of responding to nutrient availability in the context of aging is also illustrated by the observation that calorie restriction without malnutrition represents a non-genetic intervention that prolongs lifespan in many organisms. Interestingly, the ability to respond to calorie restriction resulting in lifespan increase appears linked to the ability to enter torpor (reviewed in [123]). Although most of the beneficiary effects of calorie restriction are linked to regulation of mTOR downstream of PI3K/AKT, a role for FOXO is implied through, e.g., AMPK-mediated regulation of FOXO [132,133] (reviewed in [130]).

The microbiome provides a link between nutrient intake and the host, largely controlling nutrient availability and conversion. Recently, the microbiome has become a major focus of attention as a possible contributor to a plethora of diseases including aging [134].

Several studies highlight possible interactions between FOXOs and the microbiome, and more importantly, this appears to be evolutionarily conserved as interactions are described in Hydra [135], shrimp [136], *C. elegans* [137,138], *Drosophila* [139], and humans [140]. One major function of FOXOs is to regulate transcriptional expression of antimicrobial peptides (AMPs), and this confers a resilience mechanism to maintain the native microbiome as loss of FOXO and consequent reduction of AMPs makes the gut more susceptible to colonization by foreign bacteria [135].

In contrast with the organism level, the link between resilience and resisting death at the cellular level is ambiguous and context dependent. Preventing cell death is beneficial when cells can repair the damage that results from an insult. However, if repair is insufficient or detrimental, cell clearance by induction of cell death is also beneficial as this will prevent, e.g., potential cancerous cells from proliferating. The role of FOXO activation in cell death reflects this dichotomy. FOXO activation induces expression of pro-apoptotic genes like Bcl-2-like 11 (BIM) [141] and p53 up-regulated modulator of apoptosis (PUMA) [142] and consequent cell death in cancer cells like osteosarcoma [143]. However, a cell cycle arrest induced by FOXO can also protect cells especially from insults that are accompanied by changes in cellular redox (reviewed in [131]). FOXO is in turn activated by reactive oxygen species (ROS) [144] and transcriptionally regulates proteins that protect against ROS and consequent detrimental effects of increased cellular ROS levels [145]. For example, increased DNA damage is oftentimes a consequence of increased ROS, and FOXOs have been suggested to regulate the DNA damage response at the level of ataxia-telangiectasia mutated (ATM) activity [146].

Following the description of apoptosis, several variations of programmed cell death have been described, like ferroptosis, cuproptosis, mitoptosis, paraptosis, immunogenic cell death, entosis, methuosis, parthanatos, autosis, alkaliptosis, oxeiptosis, and erebosis [147]. The relevance of these various forms of cell death to aging and age-related diseases needs to be established. However, a role for FOXOs in resilience to any of these death programs is as far as known only described for apoptosis [148] and in part for ferroptosis ([149,150]). Interestingly, in *in vitro* cell culture, increased cell density through cell-cell contact inhibition reduces ferroptosis [151]. Mechanisms involved include the Hippo pathway [152], NF2-YAP signaling [151], and lipid droplet formation [153]. High cell density down-regulates PI3K/Akt activity, followed by consequent FOXO activation and G1 cell cycle arrest. Thus, a role for FOXO in ferroptosis could be due to either FOXO inducing a G1 arrest or a G1 arrest in combination with specific non-G1-related FOXO-induced gene regulation.

Longevity and aging are tightly linked to reproduction (the *C. elegans* link between reproduction and longevity reviewed in [154]). The disposable soma theory of aging states that organisms should optimize the allocation of metabolic resources between somatic maintenance, growth, and reproduction [155–157]. As reproduction safeguards population survival, most energy will be allocated to reproduction, yet in such a way that the resources committed to maintenance are sufficient to keep the soma in good condition till at least the end of reproductive life. Allocating resources to achieve an indefinite lifespan would be illogical as death in the wild will inevitably come through predation or infectious diseases. FOXOs are described to contribute to several of the implications of this theory. For example, in *C. elegans,* insulin signaling affects both longevity and reproduction [158], and loss of germ cells extends lifespan of *C. elegans* in a manner dependent on DAF-16/FOXO (reviewed in [159]), in mammals, FOXOs contribute to the maintenance of germ cell health. Within the ovary, a pool of resting primordial follicles serves as the source of developing follicles and timely ovulation of a mature egg. Primary ovarian insufficiency is determined by exhaustion of follicles in the ovaries, and FOXO3 represses primordial follicle activation [160], and thereby ensures fertilizable ova for the entire length of female reproductive life. Oocyte-specific deletion of PTEN, a major negative regulator of PI3K, activates the entire primordial follicle pool [161], which highlights the role of PI3K/FOXO pathway in preserving reproductive longevity.

### Recent examples of resilience

Viral or bacterial infections pose a lifelong threat and are a major cause of limited lifespan. In agreement, there are multiple links between FOXOs and the function of the adaptive [162,163] and innate immune system [164], and this also holds for *C. elegans* [165].

In CAR-T cell therapy, T cells isolated from a cancer patient are used to genetically modify these cells *ex vivo* to express a chimeric antigen receptor (CAR) that targets antigen present on cancer cells. These modified cells are then infused back into the patient, where they can bind to cancer cells and trigger their destruction. Whereas this type of therapy can provide long-lasting response, it is also limited by exhaustion of these cells *in vivo*, especially in treating solid tumors. Positive treatment responses have been associated with improved T cell fitness. In line with the idea that FOXO transcriptional activity can enhance resilience of cells, it has been described recently that FOXO1 can act as a strong enhancer of CAR T cell function that boosts antitumor activity, memory, and metabolic fitness. Overexpressing FOXO1 enhances *in vivo* T cell potency and makes the cells more effective against various cancers, including solid tumors [166,167]. Interestingly, generation of CAR-T cells requires *ex vivo* activation of these cells, and this *ex vivo* activation dampens the antitumor potential of CAR-T cells. Here, FOXO1 inhibition has been described to generate CAR T cells without *ex vivo* preactivation [168]. These findings appear contradictory but may reiterate the argument we previously proposed for cancer progression that the function of FOXO is context dependent [51]. Interestingly, the precise timing of FOXO activation may profoundly shape aging trajectories and organismal resilience due to ‘transcriptional memory’. In *Drosophila melanogaster*, dFOXO activation solely in early adulthood of female fruit flies curtails later-life mortality through chromatin remodeling and Xbp1, again highlighting that timing is also an important context for FOXO activation in mediating resilience [169].

Alternatively, inhibition of FOXO was achieved by using the inhibitor AS184856 [170], and the action of this small molecule in terms of drug on- and off-target(s) is not yet clear. More importantly, using FOXO deleted cell systems, it was shown that AS184856 shows clear off-target effects [171], and in this study, an alternative inhibitor coined compound 10 (cpd10) was described. However, thus far, studies using this compound are limited (e.g., [172]), so the verdict on this small molecule is still out there. The off-target effect of AS184856 was recently confirmed, and AS184856 is described to also inhibit GSK-3 [173]. Like FOXO, GSK-3 is a substrate for the AKT/PKB kinase; furthermore, GSK-3 is a key kinase in regulating the stability of β-catenin, and thus AS184856 could be acting in some cases as a WNT mimetic [174] rather than a FOXO inhibitor. In addition, the acclaimed mode of action for AS184856 is to interfere with the DBD for DNA binding, and as discussed above, FOXOs belong to the Forkhead family characterized by a conserved DBD and mode of DNA binding, indicating the possibility that AS184856 could inhibit DNA binding and transcriptional activity of other Forkhead family members as well. Consequently, it is imperative that studies using AS184856 should be validated by siRNA or CRISPR/CAS knock-out of FOXOs. Alternatively, it shows a need for developing more specific small molecules targeting FOXOs [171].

Adult stem cells are responsible for tissue regeneration, and tissue regeneration declines with aging, thereby contributing to a decline of tissue function. The contribution of FOXOs to adult stem cell function has been well studied and examples are muscle stem cells [175], stem cells of epithelial tissues [176], hematopoietic stem cells (reviewed in [177]), neuronal stem cells [178](reviewed in [179]).

Mesenchymal stem cells (MSCs) are multipotent adult stem cells found in various tissues, including bone marrow, that can differentiate into multiple cell types, including bone, cartilage, muscle, and fat. MSCs are considered immune privileged because of their diminished expression of major histocompatibility molecules, absence of costimulatory molecules, and presence of coinhibitory molecules. Therefore, the use of allogeneic MSCs possesses broad clinical applications unconstrained by donor physiology [180]. A recent study used MSC cells genetically modified using CRISPR/CAS technology to express an active mutant of FOXO3 [181]. These cells exhibited enhanced senescence resistance, environmental resilience, and self-renewal capacity, enabling sustained tissue persistence in rodents. Compared with unmodified MSCs, transplantation of these FOXO3-modified MSCs in Cynomolgus monkeys showed numerous beneficial effects including rejuvenation as measured by two independent aging clocks.

The choice of mutations to activate FOXO3 in MSCs is noteworthy. The PKB/AKT site located in the N-terminus and the PKB/AKT site following the DBD are involved in binding to 14-3-3, and mutation of either one is sufficient to lose regulation by 14-3-3 and obtain increased nuclear location. The third PKB/AKT site is localized in the region (CR2B) that is not involved in 14-3-3 binding but in interaction with transcriptional co-factors. For example, β-catenin binding to FOXO involves CR2B, and AKT-mediated phosphorylation in this region inhibits β-catenin binding but also relieves autoinhibition [6]. Finally, with respect to the discussion of isoform specificity, it would have been informative to see whether, for example, expression of an activated FOXO1 allele in MSCs would provide similar health benefit and rejuvenation.

## Final remarks

Providing resilience to cells or even organisms can likely occur in various ways and depends on what challenge is imposed on a cell or organ. By providing resilience to cells and organs, FOXOs may influence lifespan across diverse organisms (possibly including humans). This function may be not unique to FOXOs but explains at least interest in understanding FOXO function. However, there are still important aspects to understand related to FOXOs' mode of action. If the ultimate goal will be to pharmacologically target FOXO to enhance human resilience, we need to know what FOXO isoform to target, or maybe to target them all? To understand isoform specificity, we need to know more precisely how FOXOs function as a TF. What is the role of the IDR in this, and do the IDRs determine specificity? FOXOs act in a context-specific manner, yet knowing this, we need to define in what context it would be relevant to either activate or inactivate FOXOs. This also requires strategies to develop more specific small molecules targeting FOXOs, as for now, these do not exist. Clearly, a lot of research must be done before we can manipulate FOXOs in a manner that will promote healthy aging.

## Abbreviations

DBD: DNA-binding domain
FOXO: Forkhead box O
IDRs: intrinsically disordered regions
PI3K: phosphoinositide 3-kinase
PTM: post-translational modification

## References

[1] Kops G.J.P.L. Ruiter N.D. de De Vries-Smits A.M.M. Powell D.R. Bos J.L. Burgering B.M.T 1999 Direct control of the Forkhead transcription factor AFX by protein kinase B Nature 398 630 634 10.1038/19328 10217147

[2] Brunet A. Bonni A. Zigmond M.J. Lin M.Z. Juo P. Hu L.S. et al. 1999 Akt promotes cell survival by phosphorylating and inhibiting a Forkhead transcription factor Cell 96 857 868 10.1016/s0092-8674(00)80595-4 10102273

[3] Rena G. Guo S. Cichy S.C. Unterman T.G. Cohen P 1999 Phosphorylation of the transcription factor forkhead family member FKHR by protein kinase B J. Biol. Chem. 274 17179 17183 10.1074/jbc.274.24.17179 10358075

[4] Xie J. Weiskirchen R 2020 What Does the “AKT” Stand for in the Name “AKT Kinase”? Some Historical Comments Front. Oncol. 10 1329 10.3389/fonc.2020.01329 32850422 PMC7431881

[5] Burgering B.M.Th. Coffer P.J 1995 Protein kinase B (c-Akt) in phosphatidylinositol-3-OH kinase signal transduction Nature 376 599 602 10.1038/376599a0 7637810

[6] Bourgeois B. Gui T. Hoogeboom D. Hocking H.G. Richter G. Spreitzer E et al. 2021 Multiple regulatory intrinsically disordered motifs control FOXO4 transcription factor binding and function Cell Rep 36 109446 10.1016/j.celrep.2021.109446 34320339

[7] Wang F. Marshall C.B. Yamamoto K. Li G.Y. Gasmi-Seabrook G.M.C. Okada H. et al. 2012 Structures of KIX domain of CBP in complex with two FOXO3a transactivation domains reveal promiscuity and plasticity in coactivator recruitment Proc. Natl. Acad. Sci. U.S.A. 109 6078 6083 10.1073/pnas.1119073109 22474372 PMC3341034

[8] Wang F. Marshall C.B. Yamamoto K. Li G.-Y. Plevin M.J. You H. et al. 2008 Biochemical and structural characterization of an intramolecular interaction in FOXO3a and its binding with p53 J. Mol. Biol. 384 590 603 10.1016/j.jmb.2008.09.025 18824006

[9] Bourgeois B. Spreitzer E. Platero-Rochart D. Paar M. Zhou Q. Usluer S. et al. 2025 The disordered p53 transactivation domain is the target of FOXO4 and the senolytic compound FOXO4-DRI Nat. Commun. 16 5672 10.1038/s41467-025-60844-9 40593617 PMC12216184

[10] Lin K. Hsin H. Libina N. Kenyon C 2001 Regulation of the Caenorhabditis elegans longevity protein DAF-16 by insulin/IGF-1 and germline signaling Nat. Genet. 28 139 145 10.1038/88850 11381260

[11] Paradis S. Ruvkun G 1998 Caenorhabditis elegans Akt/PKB transduces insulin receptor-like signals from AGE-1 PI3 kinase to the DAF-16 transcription factor Genes Dev. 12 2488 2498 10.1101/gad.12.16.2488 9716402 PMC317081

[12] Friedman D.B. Johnson T.E 1988 A mutation in the age-1 gene in Caenorhabditis elegans lengthens life and reduces hermaphrodite fertility Genetics 118 75 86 10.1093/genetics/118.1.75 8608934 PMC1203268

[13] Ogg S. Ruvkun G 1998 The C. elegans PTEN homolog, DAF-18, acts in the insulin receptor-like metabolic signaling pathway Mol. Cell 2 887 893 10.1016/s1097-2765(00)80303-2 9885576

[14] Dorman J.B. Albinder B. Shroyer T. Kenyon C 1995 The age-1 and daf-2 genes function in a common pathway to control the lifespan of Caenorhabditis elegans Genetics 141 1399 1406 10.1093/genetics/141.4.1399 8601482 PMC1206875

[15] Willcox B.J. Donlon T.A. He Q. Chen R. Grove J.S. Yano K. et al. 2008 FOXO3A genotype is strongly associated with human longevity Proc. Natl. Acad. Sci. U.S.A. 105 13987 13992 10.1073/pnas.0801030105 18765803 PMC2544566

[16] Huang X. Liu G. Guo J. Su Z 2018 The PI3K/AKT pathway in obesity and type 2 diabetes Int. J. Biol. Sci. 14 1483 1496 10.7150/ijbs.27173 30263000 PMC6158718

[17] Teaney N.A. Cyr N.E 2023 FoxO1 as a tissue-specific therapeutic target for type 2 diabetes Front. Endocrinol. (Lausanne) 14 1286838 10.3389/fendo.2023.1286838 37941908 PMC10629996

[18] Horvath S. Ritz B.R 2015 Increased epigenetic age and granulocyte counts in the blood of Parkinson’s disease patients Aging (Milano) 7 1130 1142 10.18632/aging.100859 PMC4712337 26655927

[19] Levine M.E. Lu A.T. Bennett D.A. Horvath S 2015 Epigenetic age of the pre-frontal cortex is associated with neuritic plaques, amyloid load, and Alzheimer’s disease related cognitive functioning Aging (Milano) 7 1198 1211 10.18632/aging.100864 PMC4712342 26684672

[20] Lu A.T. Quach A. Wilson J.G. Reiner A.P. Aviv A. Raj K. et al. 2019 DNA methylation GrimAge strongly predicts lifespan and healthspan Aging (Milano) 11 303 327 10.18632/aging.101684 30669119 PMC6366976

[21] Johnson A.A. Shokhirev M.N. Wyss-Coray T. Lehallier B 2020 Systematic review and analysis of human proteomics aging studies unveils a novel proteomic aging clock and identifies key processes that change with age Ageing Res. Rev. 60 101070 10.1016/j.arr.2020.101070 32311500

[22] Sayed N. Huang Y. Nguyen K. Krejciova-Rajaniemi Z. Grawe A.P. Gao T. et al. 2021 An inflammatory aging clock (iAge) based on deep learning tracks multimorbidity, immunosenescence, frailty and cardiovascular aging Nat. Aging 1 598 615 10.1038/s43587-021-00082-y 34888528 PMC8654267

[23] Duan R. Fu Q. Sun Y. Li Q 2022 Epigenetic clock: A promising biomarker and practical tool in aging Ageing Res. Rev. 81 101743 10.1016/j.arr.2022.101743 36206857

[24] Cockerham W.C. Yamori Y 2001 Okinawa: an exception to the social gradient of life expectancy in Japan Asia Pac. J. Clin. Nutr. 10 154 158 10.1111/j.1440-6047.2001.00232.x 11710357

[25] Willcox B.J. Tranah G.J. Chen R. Morris B.J. Masaki K.H. He Q. et al. 2016 The FoxO3 gene and cause-specific mortality Aging Cell 15 617 624 10.1111/acel.12452 27071935 PMC4933667

[26] Santo E.E. Ribel-Madsen R. Stroeken P.J. De Boer V.C.J. Hansen N.S. Commandeur M et al. 2023 FOXO3A-short is a novel regulator of non-oxidative glucose metabolism associated with human longevity Aging Cell 22 e13763 10.1111/acel.13763 36617632 PMC10014046

[27] The Emerging Risk Factors Collaboration 2011 Diabetes mellitus, fasting glucose, and risk of cause-specific death N. Engl. J. Med. 364 829 841 10.1056/NEJMoa1008862 21366474 PMC4109980

[28] Wijsman C.A. Rozing M.P. Streefland T.C.M. Le Cessie S. Mooijaart S.P. Slagboom P.E et al. 2011 Familial longevity is marked by enhanced insulin sensitivity Aging Cell 10 114 121 10.1111/j.1474-9726.2010.00650.x 21070591

[29] Li Y. Wang W.-J. Cao H. Lu J. Wu C. Hu F.-Y. et al. 2009 Genetic association of FOXO1A and FOXO3A with longevity trait in Han Chinese populations Hum. Mol. Genet. 18 4897 4904 10.1093/hmg/ddp459 19793722 PMC2790334

[30] Kleindorp R. Flachsbart F. Puca A.A. Malovini A. Schreiber S. Nebel A 2011 Candidate gene study of FOXO1, FOXO4, and FOXO6 reveals no association with human longevity in Germans Aging Cell 10 622 628 10.1111/j.1474-9726.2011.00698.x 21388494

[31] Tan Q. Soerensen M. Kruse T.A. Christensen K. Christiansen L 2013 A novel permutation test for case-only analysis identifies epistatic effects on human longevity in the FOXO gene family Aging Cell 12 690 694 10.1111/acel.12092 23607278 PMC3714332

[32] Wang M. Zhang X. Zhao H. Wang Q. Pan Y 2009 FoxO gene family evolution in vertebrates BMC Evol. Biol. 9 222 10.1186/1471-2148-9-222 19732467 PMC2746812

[33] Ohno S 1999 Gene duplication and the uniqueness of vertebrate genomes circa 1970-1999 Semin. Cell Dev. Biol. 10 517 522 10.1006/scdb.1999.0332 10597635

[34] Paik J.-H. Kollipara R. Chu G. Ji H. Xiao Y. Ding Z. et al. 2007 FoxOs are lineage-restricted redundant tumor suppressors and regulate endothelial cell homeostasis Cell 128 309 323 10.1016/j.cell.2006.12.029 17254969 PMC1855089

[35] Schmitt-Ney M 2020 The FOXO’s advantages of being a family: considerations on function and evolution Cells 9 787 10.3390/cells9030787 32214027 PMC7140813

[36] Ramaswamy S. Nakamura N. Sansal I. Bergeron L. Sellers W.R 2002 A novel mechanism of gene regulation and tumor suppression by the transcription factor FKHR Cancer Cell 2 81 91 10.1016/s1535-6108(02)00086-7 12150827

[37] Van der Vos K.E. Coffer P.J 2008 FOXO-binding partners: it takes two to tango Oncogene 27 2289 2299 10.1038/onc.2008.22 18391971

[38] Essers M.A.G. De Vries-Smits L.M.M. Barker N. Polderman P.E. Burgering B.M.T. Korswagen H.C 2005 Functional Interaction Between ß-Catenin and FOXO in Oxidative Stress Signaling Science 308 1181 1184 10.1126/science.1109083 15905404

[39] Hoogeboom D. Essers M.A.G. Polderman P.E. Voets E. Smits L.M.M. Burgering B.M.T 2008 Interaction of FOXO with beta-catenin inhibits beta-catenin/T cell factor activity J. Biol. Chem. 283 9224 9230 10.1074/jbc.M706638200 18250171

[40] Manolagas S.C. Almeida M 2007 Gone with the Wnts: beta-catenin, T-cell factor, forkhead box O, and oxidative stress in age-dependent diseases of bone, lipid, and glucose metabolism Mol. Endocrinol. 21 2605 2614 10.1210/me.2007-0259 17622581

[41] Sun X. Zhou D. Sun Y. Zhao Y. Deng Y. Pang X et al. 2024 Oxidative stress reprograms the transcriptional coactivator Yki to suppress cell proliferation Cell Rep. 43 114584 10.1016/j.celrep.2024.114584 39106181

[42] Shao D. Zhai P. Del Re D.P. Sciarretta S. Yabuta N. Nojima H. et al. 2014 A functional interaction between Hippo-YAP signalling and FoxO1 mediates the oxidative stress response Nat. Commun. 5 3315 10.1038/ncomms4315 24525530 PMC3962829

[43] Santos B.F. Grenho I. Martel P.J. Ferreira B.I. Link W 2023 FOXO family isoforms Cell Death Dis. 14 702 10.1038/s41419-023-06177-1 37891184 PMC10611805

[44] Vogel C. Marcotte E.M 2012 Insights into the regulation of protein abundance from proteomic and transcriptomic analyses Nat. Rev. Genet. 13 227 232 10.1038/nrg3185 22411467 PMC3654667

[45] Wu X. Fan Z. Chen M. Chen Y. Rong D. Cui Z. et al. 2019 Forkhead transcription factor FOXO3a mediates interferon-γ-induced MHC II transcription in macrophages Immunology 158 304 313 10.1111/imm.13116 31509237 PMC6856938

[46] Franz F. Weidinger C. Krause K. Gimm O. Dralle H. Führer D 2016 The Transcriptional Regulation of FOXO Genes in Thyrocytes Horm. Metab. Res. 48 601 606 10.1055/s-0042-105153 27258970

[47] Guertin D.A. Stevens D.M. Thoreen C.C. Burds A.A. Kalaany N.Y. Moffat J. et al. 2006 Ablation in mice of the mTORC components raptor, rictor, or mLST8 reveals that mTORC2 is required for signaling to Akt-FOXO and PKCalpha, but not S6K1 Dev. Cell 11 859 871 10.1016/j.devcel.2006.10.007 17141160

[48] Chen C.-C. Jeon S.-M. Bhaskar P.T. Nogueira V. Sundararajan D. Tonic I. et al. 2010 FoxOs inhibit mTORC1 and activate Akt by inducing the expression of Sestrin3 and Rictor Dev. Cell 18 592 604 10.1016/j.devcel.2010.03.008 20412774 PMC3031984

[49] Eijkelenboom A. Mokry M. Smits L.M. Nieuwenhuis E.E. Burgering B.M.T 2013 FOXO3 selectively amplifies enhancer activity to establish target gene regulation Cell Rep. 5 1664 1678 10.1016/j.celrep.2013.11.031 24360957

[50] Eijkelenboom A. Mokry M. Smits L.M. Polderman P.E et al. 2013 Genome-wide analysis of FOXO3 mediated transcription regulation through RNA polymerase II profiling Mol. Syst. Biol. 9 638 10.1038/msb.2012.74 23340844 PMC3564262

[51] Hornsveld M. Dansen T.B. Derksen P.W. Burgering B.M.T 2018 Re-evaluating the role of FOXOs in cancer Semin. Cancer Biol. 50 90 100 10.1016/j.semcancer.2017.11.017 29175105

[52] Hsu P. Choi E.J. Wong W.H. Lin Y.H. Vandenburgh S.A. Liu Y.C. et al. 2025 Foxo1 regulates intestinal tissue-resident memory CD8 T cell biology in an anatomic compartment- and context-specific manner Sci. Immunol. 10 eadn1894 10.1126/sciimmunol.adn1894 40215325 PMC12955362

[53] Webb A.E. Kundaje A. Brunet A 2016 Characterization of the direct targets of FOXO transcription factors throughout evolution Aging Cell 15 673 685 10.1111/acel.12479 27061590 PMC4933671

[54] Sekiya T. Muthurajan U.M. Luger K. Tulin A.V. Zaret K.S 2009 Nucleosome-binding affinity as a primary determinant of the nuclear mobility of the pioneer transcription factor FoxA Genes Dev. 23 804 809 10.1101/gad.1775509 19339686 PMC2666343

[55] Cirillo L.A. Lin F.R. Cuesta I. Friedman D. Jarnik M. Zaret K.S 2002 Opening of compacted chromatin by early developmental transcription factors HNF3 (FoxA) and GATA-4 Mol. Cell 9 279 289 10.1016/s1097-2765(02)00459-8 11864602

[56] Cirillo L.A. Zaret K.S 1999 An early developmental transcription factor complex that is more stable on nucleosome core particles than on free DNA Mol. Cell 4 961 969 10.1016/s1097-2765(00)80225-7 10635321

[57] Zaret K.S. Caravaca J.M. Tulin A. Sekiya T 2010 Nuclear mobility and mitotic chromosome binding: similarities between pioneer transcription factor FoxA and linker histone H1 Cold Spring Harb. Symp. Quant. Biol. 75 219 226 10.1101/sqb.2010.75.061 21502411

[58] Balsalobre A. Drouin J 2022 Pioneer factors as master regulators of the epigenome and cell fate Nat. Rev. Mol. Cell Biol. 23 449 464 10.1038/s41580-022-00464-z 35264768

[59] Clark K.L. Halay E.D. Lai E. Burley S.K 1993 Co-crystal structure of the HNF-3/fork head DNA-recognition motif resembles histone H5 Nature 364 412 420 10.1038/364412a0 8332212

[60] Hatta M. Liu F. Cirillo L.A 2009 Acetylation curtails nucleosome binding, not stable nucleosome remodeling, by FoxO1 Biochem. Biophys. Res. Commun. 379 1005 1008 10.1016/j.bbrc.2009.01.014 19146829

[61] Hatta M. Cirillo L.A 2007 Chromatin Opening and Stable Perturbation of Core Histone:DNA Contacts by FoxO1 Journal of Biological Chemistry 282 35583 35593 10.1074/jbc.M704735200 17923482

[62] Seoane J. Le H.-V. Shen L. Anderson S.A. Massagué J 2004 Integration of Smad and forkhead pathways in the control of neuroepithelial and glioblastoma cell proliferation Cell 117 211 223 10.1016/s0092-8674(04)00298-3 15084259

[63] Shats I. Gatza M.L. Liu B. Angus S.P. You L. Nevins J.R 2013 FOXO transcription factors control E2F1 transcriptional specificity and apoptotic function Cancer Res. 73 6056 6067 10.1158/0008-5472.CAN-13-0453 23966291 PMC3815650

[64] Nakae J. Cao Y. Hakuno F. Takemori H. Kawano Y. Sekioka R. et al. 2012 Novel repressor regulates insulin sensitivity through interaction with Foxo1 EMBO J. 31 2275 2295 10.1038/emboj.2012.97 22510882 PMC3364737

[65] Kita M. Nakae J. Kawano Y. Asahara H. Takemori H. Okado H et al. 2019 Zfp238 Regulates the thermogenic program in cooperation with foxo1 iScience 12 87 101 10.1016/j.isci.2019.01.005 30677742 PMC6352565

[66] Nemoto S. Fergusson M.M. Finkel T 2004 Nutrient availability regulates SIRT1 through a forkhead-dependent pathway Science 306 2105 2108 10.1126/science.1101731 15604409

[67] Matsuzaki H. Daitoku H. Hatta M. Aoyama H. Yoshimochi K. Fukamizu A 2005 Acetylation of Foxo1 alters its DNA-binding ability and sensitivity to phosphorylation Proc. Natl. Acad. Sci. U.S.A. 102 11278 11283 10.1073/pnas.0502738102 16076959 PMC1183558

[68] Lickwar C.R. Mueller F. Hanlon S.E. McNally J.G. Lieb J.D 2012 Genome-wide protein–DNA binding dynamics suggest a molecular clutch for transcription factor function Nature 484 251 255 10.1038/nature10985 22498630 PMC3341663

[69] Riedel C.G. Dowen R.H. Lourenco G.F. Kirienko N.V. Heimbucher T. West J.A et al. 2013 DAF-16 employs the chromatin remodeller SWI/SNF to promote stress resistance and longevity Nat. Cell Biol. 15 491 501 10.1038/ncb2720 23604319 PMC3748955

[70] Srivastava T. Diba P. Dean J.M. Banine F. Shaver D. Hagen M. et al. 2018 A TLR/AKT/FoxO3 immune tolerance-like pathway disrupts the repair capacity of oligodendrocyte progenitors J. Clin. Invest. 128 2025 2041 94158 10.1172/JCI94158 29664021 PMC5919806

[71] Langlet F. Haeusler R.A. Lindén D. Ericson E. Norris T. Johansson A et al. 2017 Selective Inhibition of FOXO1 Activator/Repressor Balance Modulates Hepatic Glucose Handling Cell 171 824 835 10.1016/j.cell.2017.09.045 29056338 PMC5687849

[72] Grimes J.A. Nielsen S.J. Battaglioli E. Miska E.A. Speh J.C. Berry D.L. et al. 2000 The co-repressor mSin3A is a functional component of the REST-CoREST repressor complex J. Biol. Chem. 275 9461 9467 10.1074/jbc.275.13.9461 10734093

[73] Liu J. Duan Z. Guo W. Zeng L. Wu Y. Chen Y. et al. 2018 Targeting the BRD4/FOXO3a/CDK6 axis sensitizes AKT inhibition in luminal breast cancer Nat. Commun. 9 5200 10.1038/s41467-018-07258-y 30518851 PMC6281582

[74] Li H. Akasaka K 2006 Conformational fluctuations of proteins revealed by variable pressure NMR Biochimica et Biophysica Acta (BBA) - Proteins and Proteomics 1764 331 345 10.1016/j.bbapap.2005.12.014 16448868

[75] Tompa P. Davey N.E. Gibson T.J. Babu M.M 2014 A million peptide motifs for the molecular biologist Mol. Cell 55 161 169 10.1016/j.molcel.2014.05.032 25038412

[76] Kumar M. Michael S. Alvarado-Valverde J. Mészáros B. Sámano-Sánchez H. Zeke A. et al. 2022 The Eukaryotic Linear Motif resource: 2022 release Nucleic Acids Res. 50 D497 D508 10.1093/nar/gkab975 34718738 PMC8728146

[77] Necci M. Piovesan D. Hoque M.T. Walsh I. Iqbal S. Vendruscolo M. et al. 2021 Critical assessment of protein intrinsic disorder prediction Nat. Methods 18 472 481 10.1038/s41592-021-01117-3 33875885 PMC8105172

[78] Xue B. Dunbrack R.L. Williams R.W. Dunker A.K. Uversky V.N 2010 PONDR-FIT: A meta-predictor of intrinsically disordered amino acids Biochimica et Biophysica Acta (BBA) - Proteins and Proteomics 1804 996 1010 10.1016/j.bbapap.2010.01.011 20100603 PMC2882806

[79] Jumper J. Evans R. Pritzel A. Green T. Figurnov M. Ronneberger O. et al. 2021 Highly accurate protein structure prediction with AlphaFold Nature 596 583 589 10.1038/s41586-021-03819-2 34265844 PMC8371605

[80] Evans R. O’Neill M. Pritzel A. Antropova N. Senior A. Green T et al. 2021 Protein complex prediction with AlphaFold-Multimer Bioinformatics 10.1101/2021.10.04.463034

[81] Gui T. Burgering B.M.T 2022 FOXOs: masters of the equilibrium FEBS J. 289 7918 7939 10.1111/febs.16221 34610198 PMC10078705

[82] Essers M.A.G. Weijzen S. De Vries-Smits A.M.M. Saarloos I. De Ruiter N.D. Bos J.L et al. 2004 FOXO transcription factor activation by oxidative stress mediated by the small GTPase Ral and JNK EMBO J 23 4802 4812 10.1038/sj.emboj.7600476 15538382 PMC535088

[83] Putker M. Madl T. Vos H.R. De Ruiter H. Visscher M. Van den Berg M.C.W et al. 2013 Redox-dependent control of FOXO/DAF-16 by transportin-1 Mol. Cell 49 730 742 10.1016/j.molcel.2012.12.014 23333309

[84] Jose E. March-Steinman W. Wilson B.A. Shanks L. Parkinson C. Alvarado-Cruz I. et al. 2024 Temporal coordination of the transcription factor response to H2O2 stress Nat. Commun. 15 3440 10.1038/s41467-024-47837-w 38653977 PMC11039679

[85] Lasick K.A. Jose E. Samayoa A.M. Shanks L. Pond K.W. Thorne C.A. et al. 2023 FOXO nuclear shuttling dynamics are stimulus-dependent and correspond with cell fate Mol. Biol. Cell 34 ar21 10.1091/mbc.E22-05-0193 36735481 PMC10011729

[86] Dansen T.B. Smits L.M.M. Van Triest M.H. De Keizer P.L.J. Van Leenen D. Koerkamp M.G et al. 2009 Redox-sensitive cysteines bridge p300/CBP-mediated acetylation and FoxO4 activity Nat. Chem. Biol 5 664 672 10.1038/nchembio.194 19648934

[87] Putker M. Vos H.R. Van Dorenmalen K. De Ruiter H. Duran A.G. Snel B et al. 2015 Evolutionary acquisition of cysteines determines FOXO paralog-specific redox signaling Antioxid. Redox Signal 22 15 28 10.1089/ars.2014.6056 25069953 PMC4270166

[88] Hu M.C.T. Lee D.F. Xia W. Golfman L.S. Ou-Yang F. Yang J.Y. et al. 2004 IκB Kinase Promotes Tumorigenesis through Inhibition of Forkhead FOXO3a Cell 117 225 237 10.1016/S0092-8674(04)00302-2 15084260

[89] Brodsky S. Jana T. Mittelman K. Chapal M. Kumar D.K. Carmi M et al. 2020 Intrinsically disordered regions direct transcription factor in vivo binding specificity Mol. Cell 79 459 471 10.1016/j.molcel.2020.05.032 32553192

[90] Crane-Robinson C. Dragan A.I. Privalov P.L 2006 The extended arms of DNA-binding domains: a tale of tails Trends Biochem. Sci. 31 547 552 10.1016/j.tibs.2006.08.006 16920361

[91] Daitoku H. Hatta M. Matsuzaki H. Aratani S. Ohshima T. Miyagishi M. et al. 2004 Silent information regulator 2 potentiates Foxo1-mediated transcription through its deacetylase activity Proc. Natl. Acad. Sci. U.S.A. 101 10042 10047 10.1073/pnas.0400593101 15220471 PMC454161

[92] Wang F. Chan C.-H. Chen K. Guan X. Lin H.-K. Tong Q 2012 Deacetylation of FOXO3 by SIRT1 or SIRT2 leads to Skp2-mediated FOXO3 ubiquitination and degradation Oncogene 31 1546 1557 10.1038/onc.2011.347 21841822

[93] Dragan A.I. Read C.M. Makeyeva E.N. Milgotina E.I. Churchill M.E.A. Crane-Robinson C. et al. 2004 DNA binding and bending by hmg boxes: energetic determinants of specificity J. Mol. Biol. 343 371 393 10.1016/j.jmb.2004.08.035 15451667

[94] Liu Y. Matthews K.S. Bondos S.E 2008 Multiple Intrinsically Disordered Sequences Alter DNA Binding by the Homeodomain of the Drosophila Hox Protein Ultrabithorax Journal of Biological Chemistry 283 20874 20887 10.1074/jbc.M800375200 18508761 PMC2475714

[95] Love J.J. Li X. Case D.A. Giese K. Grosschedl R. Wright P.E 1995 Structural basis for DNA bending by the architectural transcription factor LEF-1 Nature 376 791 795 10.1038/376791a0 7651541

[96] Alberti S. Gladfelter A. Mittag T 2019 Considerations and challenges in studying liquid-liquid phase separation and biomolecular condensates Cell 176 419 434 10.1016/j.cell.2018.12.035 30682370 PMC6445271

[97] Hyman A.A. Weber C.A. Jülicher F 2014 Liquid-Liquid Phase Separation in Biology Annu. Rev. Cell Dev. Biol. 30 39 58 10.1146/annurev-cellbio-100913-013325 25288112

[98] Banani S.F. Lee H.O. Hyman A.A. Rosen M.K 2017 Biomolecular condensates: organizers of cellular biochemistry Nat. Rev. Mol. Cell Biol. 18 285 298 10.1038/nrm.2017.7 28225081 PMC7434221

[99] Alberti S. Dormann D 2019 Liquid-Liquid phase separation in disease Annu. Rev. Genet. 53 171 194 10.1146/annurev-genet-112618-043527 31430179

[100] Brangwynne C.P. Eckmann C.R. Courson D.S. Rybarska A. Hoege C. Gharakhani J. et al. 2009 Germline P granules are liquid droplets that localize by controlled dissolution/condensation Science 324 1729 1732 10.1126/science.1172046 19460965

[101] Feric M. Vaidya N. Harmon T.S. Mitrea D.M. Zhu L. Richardson T.M et al. 2016 Coexisting liquid phases underlie nucleolar subcompartments Cell 165 1686 1697 10.1016/j.cell.2016.04.047 27212236 PMC5127388

[102] Boija A. Klein I.A. Young R.A 2021 Biomolecular Condensates and Cancer Cancer Cell 39 174 192 10.1016/j.ccell.2020.12.003 33417833 PMC8721577

[103] Hyman A.A. Weber C.A. Jülicher F 2014 Liquid-liquid phase separation in biology Annu. Rev. Cell Dev. Biol. 30 39 58 10.1146/annurev-cellbio-100913-013325 25288112

[104] Monahan Z. Ryan V.H. Janke A.M. Burke K.A. Rhoads S.N. Zerze G.H. et al. 2017 Phosphorylation of the FUS low-complexity domain disrupts phase separation, aggregation, and toxicity EMBO J. 36 2951 2967 10.15252/embj.201696394 28790177 PMC5641905

[105] Lyons H. Veettil R.T. Pradhan P. Fornero C. De La Cruz N. Ito K et al. 2023 Functional partitioning of transcriptional regulators by patterned charge blocks Cell 186 327 345 10.1016/j.cell.2022.12.013 36603581 PMC9910284

[106] Boija A. Klein I.A. Sabari B.R. Dall’Agnese A. Coffey E.L. Zamudio A.V et al. 2018 Transcription factors activate genes through the phase-separation capacity of their activation domains Cell 175 1842 1855 10.1016/j.cell.2018.10.042 30449618 PMC6295254

[107] Chong S. Dugast-Darzacq C. Liu Z. Dong P. Dailey G.M. Cattoglio C. et al. 2018 Imaging dynamic and selective low-complexity domain interactions that control gene transcription Science 361 eaar2555 10.1126/science.aar2555 29930090 PMC6961784

[108] Zamudio A.V. Dall’Agnese A. Henninger J.E. Manteiga J.C. Afeyan L.K. Hannett N.M et al. 2019 Mediator condensates localize signaling factors to key cell identity genes Mol. Cell 76 753 766 10.1016/j.molcel.2019.08.016 31563432 PMC6898777

[109] Sabari B.R. Dall’Agnese A. Boija A. Klein I.A. Coffey E.L. Shrinivas K. et al. 2018 Coactivator condensation at super-enhancers links phase separation and gene control Science 361 eaar3958 10.1126/science.aar3958 29930091 PMC6092193

[110] Cho W.K. Spille J.H. Hecht M. Lee C. Li C. Grube V. et al. 2018 Mediator and RNA polymerase II clusters associate in transcription-dependent condensates Science 361 412 415 10.1126/science.aar4199 29930094 PMC6543815

[111] Guo Y.E. Manteiga J.C. Henninger J.E. Sabari B.R. Dall’Agnese A. Hannett N.M. et al. 2019 Pol II phosphorylation regulates a switch between transcriptional and splicing condensates Nature 572 543 548 10.1038/s41586-019-1464-0 31391587 PMC6706314

[112] Henninger J.E. Oksuz O. Shrinivas K. Sagi I. LeRoy G. Zheng M.M et al. 2021 RNA-Mediated feedback control of transcriptional condensates Cell 184 207 225 10.1016/j.cell.2020.11.030 33333019 PMC8128340

[113] Quail T. Golfier S. Elsner M. Ishihara K. Murugesan V. Renger R. et al. 2021 Force generation by protein–DNA co-condensation Nat. Phys. 17 1007 1012 10.1038/s41567-021-01285-1

[114] Boehning M. Dugast-Darzacq C. Rankovic M. Hansen A.S. Yu T. Marie-Nelly H. et al. 2018 RNA polymerase II clustering through carboxy-terminal domain phase separation Nat. Struct. Mol. Biol. 25 833 840 10.1038/s41594-018-0112-y 30127355

[115] Lu H. Yu D. Hansen A.S. Ganguly S. Liu R. Heckert A. et al. 2018 Phase-separation mechanism for C-terminal hyperphosphorylation of RNA polymerase II Nature 558 318 323 10.1038/s41586-018-0174-3 29849146 PMC6475116

[116] Wei M.-T. Chang Y.-C. Shimobayashi S.F. Shin Y. Strom A.R. Brangwynne C.P 2020 Nucleated transcriptional condensates amplify gene expression Nat. Cell Biol. 22 1187 1196 10.1038/s41556-020-00578-6 32929202

[117] Gui T. Fleming C. Manzato C. Bourgeois B. Sirati N. Heuer J et al. 2023 Targeted perturbation of signaling-driven condensates Mol. Cell 83 4141 4157 10.1016/j.molcel.2023.10.023 37977121

[118] Ordóñez-Morán P. Irmisch A. Barbáchano A. Chicote I. Tenbaum S. Landolfi S. et al. 2014 SPROUTY2 is a β-catenin and FOXO3a target gene indicative of poor prognosis in colon cancer Oncogene 33 1975 1985 10.1038/onc.2013.140 23624922

[119] McSwiggen D.T. Mir M. Darzacq X 2019 Evaluating phase separation in live cells: diagnosis, caveats, and functional consequences Genes Dev 33 1619 1634 10.1101/gad.331520.119 31594803 PMC6942051

[120] Eijkelenboom A. Burgering B.M.T 2013 FOXOs: signalling integrators for homeostasis maintenance Nat. Rev. Mol. Cell Biol. 14 83 97 10.1038/nrm3507 23325358

[121] Medema R.H. Jäättelä M 2010 Cytosolic FoxO1: alive and killing Nat. Cell Biol. 12 642 643 10.1038/ncb0710-642 20596046

[122] Grossi V. Fasano C. Celestini V. Lepore Signorile M. Sanese P. Simone C 2019 Chasing the FOXO3: Insights into its new mitochondrial lair in colorectal cancer landscape Cancers (Basel) 11 414 10.3390/cancers11030414 30909600 PMC6468785

[123] Wheatley W.S.R. Marshall C.J. Taddei L. Hitrec T. Pickering A.E. Ambler M.T 2025 The cold truth: torpor as a confound in studies of caloric restriction J. Comp. Physiol. B, Biochem. Syst. Environ. Physiol. 195 263 276 10.1007/s00360-025-01616-1 40488879 PMC12289793

[124] Heldmaier G. Ortmann S. Elvert R 2004 Natural hypometabolism during hibernation and daily torpor in mammals Respir. Physiol. Neurobiol. 141 317 329 10.1016/j.resp.2004.03.014 15288602

[125] Brown J.C.L. Staples J.F 2010 Mitochondrial metabolism during fasting-induced daily torpor in mice Biochimica et Biophysica Acta (BBA) - Bioenergetics 1797 476 486 10.1016/j.bbabio.2010.01.009 20080074

[126] Horikawa M. Fukuyama M. Antebi A. Mizunuma M 2024 Regulatory mechanism of cold-inducible diapause in Caenorhabditis elegans Nat. Commun. 15 5793 10.1038/s41467-024-50111-8 38987256 PMC11237089

[127] Kloet D.E.A. Burgering B.M.T 2011 The PKB/FOXO switch in aging and cancer Biochimica et Biophysica Acta (BBA) - Molecular Cell Research 1813 1926 1937 10.1016/j.bbamcr.2011.04.003 21539865

[128] Henderson S.T. Johnson T.E 2001 daf-16 integrates developmental and environmental inputs to mediate aging in the nematode Caenorhabditis elegans Curr. Biol. 11 1975 1980 10.1016/s0960-9822(01)00594-2 11747825

[129] Zhang X. Ge L. Jin G. Liu Y. Yu Q. Chen W. et al. 2024 Cold-induced FOXO1 nuclear transport aids cold survival and tissue storage Nat. Commun. 15 2859 10.1038/s41467-024-47095-w 38570500 PMC10991392

[130] Greer E.L. Banko M.R. Brunet A 2009 AMP-activated protein kinase and FoxO transcription factors in dietary restriction-induced longevity Ann. N. Y. Acad. Sci. 1170 688 692 10.1111/j.1749-6632.2009.04019.x 19686213 PMC2814416

[131] Klotz L.-O. Sánchez-Ramos C. Prieto-Arroyo I. Urbánek P. Steinbrenner H. Monsalve M 2015 Redox regulation of FoxO transcription factors Redox Biol. 6 51 72 10.1016/j.redox.2015.06.019 26184557 PMC4511623

[132] Barthel A. Schmoll D. Krüger K.D. Roth R.A. Joost H.G 2002 Regulation of the Forkhead Transcription Factor FKHR (FOXO1a) by Glucose Starvation and AICAR, an Activator of AMP-Activated Protein Kinase Endocrinology 143 3183 3186 10.1210/endo.143.8.8792 12130586

[133] Greer E.L. Oskoui P.R. Banko M.R. Maniar J.M. Gygi M.P. Gygi S.P. et al. 2007 The energy sensor AMP-activated protein kinase directly regulates the mammalian FOXO3 transcription factor J. Biol. Chem. 282 30107 30119 10.1074/jbc.M705325200 17711846

[134] Finlay B.B. Pettersson S. Melby M.K. Bosch T.C.G 2019 The microbiome mediates environmental effects on aging Bioessays 41 e1800257 10.1002/bies.201800257 31157928

[135] Mortzfeld B.M. Taubenheim J. Fraune S. Klimovich A.V. Bosch T.C.G 2018 Stem Cell transcription factor foxo controls microbiome resilience in *Hydra* Front. Microbiol. 9 629 10.3389/fmicb.2018.00629 29666616 PMC5891625

[136] Li C. Hong P.P. Yang M.C. Zhao X.F. Wang J.X 2021 FOXO regulates the expression of antimicrobial peptides and promotes phagocytosis of hemocytes in shrimp antibacterial immunity PLoS Pathog. 17 e1009479 10.1371/journal.ppat.1009479 33798239 PMC8046353

[137] Zhang F. Weckhorst J.L. Assié A. Hosea C. Ayoub C.A. Khodakova A.S. et al. 2021 Natural genetic variation drives microbiome selection in the Caenorhabditis elegans gut Curr. Biol. 31 2603 2618 10.1016/j.cub.2021.04.046 34048707 PMC8222194

[138] Urrutia A. García-Angulo V.A. Fuentes A. Caneo M. Legüe M. Urquiza S. et al. 2020 Bacterially produced metabolites protect C. elegans neurons from degeneration PLoS Biol. 18 e3000638 10.1371/journal.pbio.3000638 32208418 PMC7092960

[139] Fink C. Hoffmann J. Knop M. Li Y. Isermann K. Roeder T 2016 Intestinal FoxO signaling is required to survive oral infection in Drosophila Mucosal Immunol. 9 927 936 10.1038/mi.2015.112 26627459

[140] Yao Y. Kim G. Shafer S. Chen Z. Kubo S. Ji Y et al. 2022 Mucus sialylation determines intestinal host-commensal homeostasis Cell 185 1172 1188 10.1016/j.cell.2022.02.013 35303419 PMC9088855

[141] Gilley J. Coffer P.J. Ham J 2003 FOXO transcription factors directly activate bim gene expression and promote apoptosis in sympathetic neurons J. Cell Biol. 162 613 622 10.1083/jcb.200303026 12913110 PMC2173804

[142] You H. Pellegrini M. Tsuchihara K. Yamamoto K. Hacker G. Erlacher M. et al. 2006 FOXO3a-dependent regulation of Puma in response to cytokine/growth factor withdrawal J. Exp. Med. 203 1657 1663 10.1084/jem.20060353 16801400 PMC2118330

[143] Oyama T. Brashears C.B. Rathore R. Benect-Hamilton H. Caldwell K.E. Dirckx N. et al. 2025 PHGDH inhibition and FOXO3 modulation drives PUMA-dependent apoptosis in osteosarcoma Cell Death Dis. 16 89 10.1038/s41419-025-07378-6 39934141 PMC11814296

[144] Kops G.J.P.L. Dansen T.B. Polderman P.E. Saarloos I. Wirtz K.W.A. Coffer P.J. et al. 2002 Forkhead transcription factor FOXO3a protects quiescent cells from oxidative stress Nature 419 316 321 10.1038/nature01036 12239572

[145] Ferber E.C. Peck B. Delpuech O. Bell G.P. East P. Schulze A 2012 FOXO3a regulates reactive oxygen metabolism by inhibiting mitochondrial gene expression Cell Death Differ. 19 968 979 10.1038/cdd.2011.179 22139133 PMC3354049

[146] Tsai W.-B. Chung Y.M. Takahashi Y. Xu Z. Hu M.C.-T 2008 Functional interaction between FOXO3a and ATM regulates DNA damage response Nat. Cell Biol. 10 460 467 10.1038/ncb1709 18344987 PMC2674111

[147] Park W. Wei S. Kim B.-S. Kim B. Bae S.-J. Chae Y.C. et al. 2023 Author Correction: Diversity and complexity of cell death: a historical review Exp. Mol. Med. 55 2083 10.1038/s12276-023-01107-9 37731035 PMC10545689

[148] Zhang X. Tang N. Hadden T.J. Rishi A.K 2011 Akt, FoxO and regulation of apoptosis Biochimica et Biophysica Acta (BBA) - Molecular Cell Research 1813 1978 1986 10.1016/j.bbamcr.2011.03.010 21440011

[149] Zhong S. Chen W. Wang B. Gao C. Liu X. Song Y. et al. 2023 Energy stress modulation of AMPK/FoxO3 signaling inhibits mitochondria-associated ferroptosis Redox Biol. 63 102760 10.1016/j.redox.2023.102760 37267686 PMC10244700

[150] Huang H. Smits A.M.M. Gulersonmez C. Stigter E. Dansen T.B et al. 2025 Activation of a FOXO3-induced cell cycle arrest regulates ferroptosis Cell Death Discov. 11 465 10.1038/s41420-025-02760-x 41102177 PMC12533257

[151] Wu J. Minikes A.M. Gao M. Bian H. Li Y. Stockwell B.R. et al. 2019 Publisher Correction: Intercellular interaction dictates cancer cell ferroptosis via NF2-YAP signalling Nature 572 E20 10.1038/s41586-019-1480-0 31371811

[152] Yang W.-H. Ding C.-K.C. Sun T. Rupprecht G. Lin C.-C. Hsu D et al. 2019 The Hippo Pathway Effector TAZ Regulates Ferroptosis in Renal Cell Carcinoma Cell Rep 28 2501 2508 10.1016/j.celrep.2019.07.107 31484063 PMC10440760

[153] Lee H. Horbath A. Kondiparthi L. Meena J.K. Lei G. Dasgupta S. et al. 2024 Cell cycle arrest induces lipid droplet formation and confers ferroptosis resistance Nat. Commun. 15 79 10.1038/s41467-023-44412-7 38167301 PMC10761718

[154] Luo S. Murphy C.T 2011 Caenorhabditis elegans reproductive aging: Regulation and underlying mechanisms Genesis 49 53 65 10.1002/dvg.20694 21105070

[155] Kirkwood T.B.L. Holliday R 1979 The evolution of ageing and longevity Proc. R. Soc. Lond., B, Biol. Sci. 205 531 546 10.1098/rspb.1979.0083 42059

[156] Kirkwood T.B.L 2005 Understanding the odd science of aging Cell 120 437 447 10.1016/j.cell.2005.01.027 15734677

[157] Drenos F. Kirkwood T.B.L 2005 Modelling the disposable soma theory of ageing Mech. Ageing Dev. 126 99 103 10.1016/j.mad.2004.09.026 15610767

[158] Tissenbaum H.A. Ruvkun G 1998 An insulin-like signaling pathway affects both longevity and reproduction in caenorhabditis elegans Genetics 148 703 717 10.1093/genetics/148.2.703 9504918 PMC1459840

[159] Kenyon C 2010 A pathway that links reproductive status to lifespan in Caenorhabditis elegans Ann. N. Y. Acad. Sci. 1204 156 162 10.1111/j.1749-6632.2010.05640.x 20738286

[160] Castrillon D.H. Miao L. Kollipara R. Horner J.W. DePinho R.A 2003 Suppression of ovarian follicle activation in mice by the transcription factor Foxo3a Science 301 215 218 10.1126/science.1086336 12855809

[161] Reddy P. Liu L. Adhikari D. Jagarlamudi K. Rajareddy S. Shen Y et al. 2008 Oocyte-specific deletion of Pten causes premature activation of the primordial follicle pool Science 319 611 613 10.1126/science.1152257 18239123

[162] Amin R.H. Schlissel M.S 2008 Foxo1 directly regulates the transcription of recombination-activating genes during B cell development Nat. Immunol. 9 613 622 10.1038/ni.1612 18469817 PMC2612116

[163] Kerdiles Y.M. Beisner D.R. Tinoco R. Dejean A.S. Castrillon D.H. DePinho R.A. et al. 2009 Foxo1 links homing and survival of naive T cells by regulating L-selectin, CCR7 and interleukin 7 receptor Nat. Immunol. 10 176 184 10.1038/ni.1689 19136962 PMC2856471

[164] Becker T. Loch G. Beyer M. Zinke I. Aschenbrenner A.C. Carrera P. et al. 2010 FOXO-dependent regulation of innate immune homeostasis Nature 463 369 373 10.1038/nature08698 20090753

[165] Singh V. Aballay A 2009 Regulation of DAF-16-mediated Innate Immunity in Caenorhabditis elegans J. Biol. Chem. 284 35580 35587 10.1074/jbc.M109.060905 19858203 PMC2790988

[166] Chan J.D. Scheffler C.M. Munoz I. Sek K. Lee J.N. Huang Y.-K. et al. 2024 FOXO1 enhances CAR T cell stemness, metabolic fitness and efficacy Nature 629 201 210 10.1038/s41586-024-07242-1 38600376 PMC11062918

[167] Doan A.E. Mueller K.P. Chen A.Y. Rouin G.T. Chen Y. Daniel B. et al. 2024 Publisher Correction: FOXO1 is a master regulator of memory programming in CAR T cells Nature 629 E11 E11 E11 10.1038/s41586-024-07450-9 38654101 PMC11078720

[168] Marchais M. Simula L. Phayanouvong M. Mami-Chouaib F. Bismuth G. Decroocq J. et al. 2023 FOXO1 Inhibition Generates Potent Nonactivated CAR T Cells against Solid Tumors Cancer Immunol. Res. 11 1508 1523 10.1158/2326-6066.CIR-22-0533 37649096

[169] Martínez Corrales G. Li M. Svermova T. Goncalves A. Voicu D. Dobson A.J. et al. 2022 Transcriptional memory of dFOXO activation in youth curtails later-life mortality through chromatin remodeling and Xbp1 Nat. Aging 2 1176 1190 10.1038/s43587-022-00312-x 37118537 PMC7614430

[170] Nagashima T. Shigematsu N. Maruki R. Urano Y. Tanaka H. Shimaya A. et al. 2010 Discovery of novel forkhead box O1 inhibitors for treating type 2 diabetes: improvement of fasting glycemia in diabetic db/db mice Mol. Pharmacol. 78 961 970 10.1124/mol.110.065714 20736318

[171] Lee Y.-K. Diaz B. Deroose M. Lee S.X. Belvedere S. Accili D. et al. 2021 FOXO1 inhibition synergizes with FGF21 to normalize glucose control in diabetic mice Mol. Metab. 49 101187 10.1016/j.molmet.2021.101187 33577983 PMC7966865

[172] Jang J.Y. Hwang I. Pan H. Yao J. Alinari L. Imada E. et al. 2022 A FOXO1-dependent transcription network is a targetable vulnerability of mantle cell lymphomas J. Clin. Invest. 132 e160767 10.1172/JCI160767 36282572 PMC9753996

[173] He D.L. Zhang X.Y. Su J.Y. Zhang Q. Zhao L.X. Wu T.Y. et al. 2025 Identification of AS1842856 as a novel small-molecule GSK3α/β inhibitor against Tauopathy by accelerating GSK3α/β exocytosis Aging Cell 24 e14336 10.1111/acel.14336 39287420 PMC11709109

[174] Ketzer F. Büttner U. Geist D. Kick A. Wirth T. Ushmorov A 2025 Beyond FOXO1: AS1842856 inhibits GSK3 to enhance cytotoxic effects in B-ALL Blood Adv. 9 3441 3454 10.1182/bloodadvances.2024015560 40638129 PMC12274837

[175] García-Prat L. Perdiguero E. Alonso-Martín S. Dell’Orso S. Ravichandran S. Brooks S.R. et al. 2020 FoxO maintains a genuine muscle stem-cell quiescent state until geriatric age Nat. Cell Biol. 22 1307 1318 10.1038/s41556-020-00593-7 33106654

[176] Ludikhuize M.C. Meerlo M. Gallego M.P. Xanthakis D. Burgaya Julià M. Nguyen N.T.B et al. 2020 Mitochondria define intestinal stem cell differentiation downstream of a FOXO/Notch Axis Cell Metab 32 889 900 10.1016/j.cmet.2020.10.005 33147486

[177] Tothova Z. Gilliland D.G 2007 FoxO transcription factors and stem cell homeostasis: insights from the hematopoietic system Cell Stem Cell 1 140 152 10.1016/j.stem.2007.07.017 18371346

[178] Renault V.M. Rafalski V.A. Morgan A.A. Salih D.A.M. Brett J.O. Webb A.E. et al. 2009 FoxO3 regulates neural stem cell homeostasis Cell Stem Cell 5 527 539 10.1016/j.stem.2009.09.014 19896443 PMC2775802

[179] Santo E.E. Paik J 2018 FOXO in Neural Cells and Diseases of the Nervous System Curr Top Dev Biol 127 105 118 10.1016/bs.ctdb.2017.10.002 29433734 PMC5881381

[180] Li Y. Jin M. Guo D. Shen S. Lu K. Pan R. et al. 2024 Unveiling the immunogenicity of allogeneic mesenchymal stromal cells: Challenges and strategies for enhanced therapeutic efficacy Biomedicine & Pharmacotherapy 180 117537 10.1016/j.biopha.2024.117537 39405918

[181] Lei J. Xin Z. Liu N. Ning T. Jing Y. Qiao Y. et al. 2025 Senescence-resistant human mesenchymal progenitor cells counter aging in primates Cell 188 5039 5061 10.1016/j.cell.2025.05.021 40516525

